# Mechanism of Qiling Fuzheng Qingjie granules in alleviating doxorubicin-induced T cell immune dysfunction via mitochondrial energy metabolism

**DOI:** 10.1186/s13020-026-01405-0

**Published:** 2026-05-19

**Authors:** Ruiming Yang, Jie Yuan, Ruihan Sun, Shunyong Wang, Zewei Zhuo, Xiaoyuan Zhang, Shuyou Chen, Junying Guo, Lisheng You, Zhiyun Cao, Xuzheng Chen, Haiying Fu

**Affiliations:** 1 Academy of Integrative Medicine, Fujian University of Traditional Chinese Medicine, Fuzhou, 350122 Fujian China; 2 College of Integrative Medicine, Fujian University of Traditional Chinese Medicine, Fuzhou, 350122 Fujian China; 3 Fujian Key Laboratory of Integrative Medicine on Geriatrics, Fuzhou, Fujian 350122 People’s Republic of China; 4 The Third People’s Hospital Affiliated to Fujian University of Traditional Chinese Medicine, Fuzhou, 350001 Fujian China; 5 Department of Laboratory Medicine, Fujian Cancer Hospital, Fuzhou, 350014 Fujian China; 6 Department of Pathology, Fuzhou University Affiliated Provincial Hospital, Fuzhou, 350001 Fujian China; 7 Shengli Clinical Medical College of Fujian Medical University, Fuzhou, 350001 Fujian China

**Keywords:** Qiling Fuzheng Qingjie granules, Doxorubicin, T lymphocyte subsets, Mitochondrial energy metabolism

## Abstract

This study investigates the mechanism by which Qiling Fuzheng Qingjie Granule (QFQ) regulates immune function following doxorubicin (DOX) chemotherapy. Network pharmacology analysis was employed to explore the relationship between QFQ and the immune system. A mouse model of DOX-induced immune dysfunction was established and treated with QFQ. Techniques including histological staining, peripheral complete blood count analysis, flow cytometry, and seahorse metabolic analysis were used to systematically evaluate the effects of QFQ on immune organs, bone marrow hematopoiesis, T-cell subset distribution and function, mitochondrial metabolism, and antitumor efficacy. The results demonstrated that QFQ significantly alleviated DOX-induced atrophy of the thymus and spleen, preserved bone marrow hematopoietic function, and reduced peripheral blood cell loss. Mechanistically, QFQ improved mitochondrial membrane potential and oxidative phosphorylation, thereby restoring T-cell metabolic capacity, enhancing T-cell activation and proliferation, and restoring cytotoxic T lymphocyte (CTL) function. Consequently, QFQ rebalanced the dynamics among Th1/Th2/Th17/Treg, Effector Memory T/Effector T cells (Tem/Teff), and Central Memory T cells (Tcm) subsets, ameliorated DOX-induced immune dysregulation. In addition, QFQ intervention not only enhanced antitumor efficacy in the A20 lymphoma mouse model but also significantly alleviated the exhaustion phenotype in CD8^+^T cells. In summary, this investigation establishes that QFQ enhances T-cell functional performance primarily through the regulation of mitochondrial energy metabolism. By restoring metabolic homeostasis in T lymphocytes, QFQ effectively counteracts DOX-induced immune dysfunction, highlighting its potential as a promising adjunctive therapy in chemotherapy-induced immunosuppression.

**Supplementary Information:**

The online version contains supplementary material available at 10.1186/s13020-026-01405-0.

## Introduction

Chemotherapy, a common tumor treatment, frequently induces adverse effects such as bone marrow suppression and immunosuppression, often leading to treatment interruption or dose reduction, and increasing risks of infection and tumor recurrence [1–6]. Although low-dose cytotoxic agents can promote immune cell infiltration and enhance antitumor immunity [7–11]. Near-maximum tolerated doses or repeated cycles often cause immunosuppression—especially impairing T cell-mediated immunity by reducing T cell subsets, blocking activation, and inducing senescence—thereby contributing to tumor recurrence [12, 13]. For example, doxorubicin (DOX), a primary chemotherapeutic drug for malignancies such as lymphoma and breast cancer, can elicit immune responses in the initial phases of treatment by augmenting antigen presentation and improving antitumor immunological effects [14, 15]. Nonetheless, with successive treatment cycles, DOX progressively demonstrates considerable immunosuppressive effects, which are closely associated with its influence on T cell senescence or dysfunction [16], despite the clinical development of novel formulations such as liposomes [17] and nanoparticles [18].

T cell metabolic reprogramming is a critical driver of chemotherapy-induced T cell impairment [19]. A metabolic change from mitochondrial oxidative phosphorylation (OXPHOS) to aerobic glycolysis occurs when T cells go from a resting to an active state. In order to sustain T cell activity and promote efficient immunological responses, mitochondrial OXPHOS is essential [20, 21]. According to studies, chemotherapy can harm the mitochondria of T cells, resulting in a decrease in the potential of the mitochondrial membrane and the depletion of different mitochondrial stores. This ultimately jeopardizes the energy supply and metabolic capacity of T cells, leading to a decline in T cell numbers and function as well as a drop in ATP synthesis [22]. Consequently, T cells block the metabolic reprogramming required for T cell activation by failing to quickly transition from a mitochondrial oxidative energy state to a glycolysis-dependent metabolic state when encountering an antigen [23]. Thus, it may be possible to successfully restore T cell activity and has great potential to alleviate T cell immunosuppression in cancer patients after chemotherapy by restoring mitochondrial metabolic function in T cells. Qiling Fuzheng Qingjie Granule (QFQ) is a compound formulation in Traditional Chinese Medicine, categorized as a treatment for reinforcing healthy Qi and tonifying the body. It functions to replenish Qi and nourish Yin, strengthen constitutional resilience, eliminate pathogenic factors, clear heat, and detoxify. This formula is composed of medicinal ingredients including *Astragalus membranaceus* (Fisch.) Bunge*, Ganoderma lucidum*(Leyss. ex Fr.) Karst*, Prunella vulgaris* L.*, Dioscorea opposita* Thunb*., Hedyotis diffusa* (Willd.) Roxb. and *Ligustrum lucidum* Ait. It is an in-hospital preparation of the Second Affiliated Hospital of Fujian University of Traditional Chinese Medicine. QFQ is primarily used in cancer patients during the perioperative, chemotherapy, and radiotherapy periods. Basic research shows that QFQ induces tumor cell apoptosis via mitochondria-dependent pathways, suppresses tumor growth by modulating glycolysis-related proteins and improving energy metabolism, and enhances CD3⁺ and CD4⁺T cell proportions in orthotopic liver cancer mice [24]. It also counteracts CTX-induced weight loss, thymus atrophy, leukopenia, thrombocytopenia, anemia, and other immunological dysfunctions. Furthermore, QFQ elevates serum TNF-α and IL-2 levels, restores T lymphocyte subset balance, and enhances chemotherapeutic efficacy [25]. These findings support QFQ’s antitumor and immunomodulatory potential; however, its impact on chemotherapy-induced immune dysregulation from the perspective of mitochondrial energy metabolism remains unexplored.

The objective of this study was to clarify the putative immunomodulatory functions of QFQ by identifying and analyzing its potential targets and signaling pathways associated with immune regulation using a network pharmacology method. To systematically assess the effects of QFQ on bone marrow suppression DOX-induced thymic and splenic atrophy, and the distribution and dynamic changes of T lymphocyte subsets, such as Th1, Th2, Th17, Treg cells, Tn (Naïve T cells, Tn), Tem ( Effector Memory T cells, Tem) / Teff (Effector T cells, Teff), and Tcm (Central Memory T cells, Tcm), a DOX-induced chemotherapy mouse model was developed. QFQ's regulatory effects on T cell activation, proliferation, and CTL activity are explored as well in this work. Furthermore, the impact of QFQ on T cell dysfunction after DOX treatment is investigated from the standpoint of mitochondrial energy metabolism.

## Materials and methods

### Reagents

QFQ (Cat#102310006) was provided by the Preparation Room of the Second Affiliated Hospital of Fujian University of Traditional Chinese Medicine (Fuzhou, China). Doxorubicin (DOX, Cat#H33021980) was obtained from Hanhui Pharmaceutical Co., LTD (Hangzhou, China), L/D-Fixable Viability Stain 700 (Cat#564997), CD3e-PE-CF594 (Cat#562286), CD8a-BV510 (Cat#563068), CD62L-PE (Cat#553151), CD45R/B220-BV786 (Cat#563894), CD44-Percp-Cy5.5 (Cat#560570), Ly-6G-BV650 (Cat#740554), Ly-6C-BV421 (Cat#562727), CD11b-FITC (Cat#557396), IFN-γ-BV480 (Cat#566097), IL-17A-Alexa Fluor® 647 (Cat#560184), Purified NA/LE Hamster Anti-Mouse CD3e (145-2C11) (Cat#553057), Purified NA/LE Hamster Anti-Mouse CD28 (37.51) (Cat#553294), PD-1-BV421 (Cat#562584), CD28-PE (Cat#553297), CD152-Percp-cy5.5 (Cat#106316), IL-4-PE (Cat#554435), CD25-BB515 (Cat#564424), CD4-BV605 (Cat#563151), BD Fixation/Permeabilization Kit (Cat#554714), Foxp3-PE (Cat#563101), Leukocyte Activation Cocktail (Cat#550583), CFSE-FITC (Cat#565082), Stain Buffer (FBS) (Cat#554656) were purchased from BD Corporation (NJ, USA). Perforin-APC (Cat#154304) was obtained from Anolun Biotechnology Co., LTD (Beijing, China). KLRG1-BV786 (Cat#78-5893-82) was purchased from Thermo Fisher Scientific (MA, USA). Reticulocyte Stain (Cat#G1400), Mitochondrial deep red fluorescence staining kit (Cat#C1997S) was purchased from Beyotime Biotechnology Co., LTD (Shanghai, China). JC-1 kit (Cat#KGA1904) was obtained by KeyGEN Biotechnology Co., LTD (Jiangsu, China). Seahorse XF Real-time ATP Rate Assay Kit (Cat#103592-100), Seahorse XF Cell Mitochondrial Stress Assay Kit (Cat#103015-100), Seahorse XF Glycolysis Rate Assay Kit (Cat#103344-100) was purchased at Agilent Technologies Co., LTD (Beijing, China). DNase I (Cat#D8071) and Collagenase Type I (Cat#C8140) were purchased at Solarbio Science & Technology Co., Ltd. (Beijing, China).

### Identification of targets for QFQ therapy within the immune system and performing analyses utilizing the Kyoto Encyclopedia of Genes and Genomes (KEGG) and Gene Ontology (GO)

Using the names of the six components of QFQ as keywords, we searched the Traditional Chinese Medicine Systems Pharmacology (TCMSP) database (https://old.tcmsp-e.com/tcmsp.php). Compounds were screened based on the following criteria: oral bioavailability (OB) > 30% and drug-likeness (DL) ≥ 0.18. The corresponding SMILES identifiers were retrieved from the PubChem database (https://pubchem.ncbi.nlm.nih.gov) based on the ingredient names. These identifiers were then used to predict potential targets of the compounds using the SwissTargetPrediction database (http://www.swisstargetprediction.ch). We then conducted a search using “Immune response” as a keyword in GeneCards database (https://www.genecards.org). The Venny 2.1 software (https://bioinfogp.cnb.csic.es/tools/venny) was used to determine the shared QFQ and immune response-related targets. To explore the connections and identify key target genes among intersecting targets involved in the therapeutic effects of QFQ on immune response, a PPI network was built utilizing the STRING database (https://cn.string-db.org). The PPI network obtained from the STRING database was visualized using Cytoscape 3.9.1. Using the Centiscape 2.2 plugin, the network's nodes were evaluated for Degree, Betweenness unDir, and Closeness unDir. To gain a deeper understanding of the interaction between target genes and diseases, as well as the underlying mechanisms of QFQ, the Metascape database (https://metascape.org/gp/index.html) was used to perform GO functional enrichment analysis and KEGG analysis (https://www.kegg.jp) on the key target genes. Use the online drawing tool (https://www.bioinformatics.com.cn/) to create a bar chart.

### Preparation of QFQ and animals grouping and treatment

A total of 48 specific-pathogen-free (SPF) male BALB/c mice (7 weeks old; 20–22 g) were obtained from SLAC Laboratory Animal Technology Co., Ltd. (Shanghai, China). The animals were maintained under standard conditions with controlled temperature (22–26 °C) and humidity (30–50%). All experimental procedures were approved by the Animal Care and Use Committee of Fujian University of Traditional Chinese Medicine (Approval No. FJTCMIACUC2023085). Mice were randomly assigned to four groups (n = 12): the Control group, the DOX group, the QFQ group, and the DOX + QFQ group. The administration regimen was as follows: The DOX group received DOX (5 mg/kg) via intravenous injection every 5 days for a total of three injections. The QFQ group was administered QFQ (4.5 g/kg) by oral gavage daily for 22 days. The DOX + QFQ group received both the same DOX regimen as the DOX group and the daily QFQ treatment as the QFQ group. The Control group was given an equal volume of saline orally. On the seventh day following the last DOX injection, tissue was collected and pertinent markers were assessed. For specific drug administration procedures, refer to Fig. 2A.

### Measurement of body weight and assessment of thymus and spleen indexes

Mouse general condition and body weight were regularly monitored to generate a growth curve. Following euthanasia, the thymus and spleen were collected, photographed, and weighed. The thymus and spleen indexes were calculated for each mouse using the following formulas: Spleen index (%) = (spleen weight/body weight) × 100. Thymus index (%) = (thymus weight/body weight) × 100.

### Hematological analysis

Blood samples were collected for conducting a complete blood count and assessing the reticulocyte ratio. The parameters of the complete blood count were analyzed by the Third People's Hospital of Fujian Province. The percentage of reticulocytes was observed under an Leica DM4000B inverted microscope (Leica, Wetzlar, Germany) using Brilliant Cresyl Blue staining solution. The reticulocyte ratio (%) for each mouse was calculated using the formula: Reticulocyte percentage (%) = (number of reticulocytes/total number of red blood cells) × 100.

### Wright-Giemsa staining

Muscle tissue was removed from each mouse femur, and both epiphyses were excised. The marrow was then flushed from the bone cavity onto a glass slide using a syringe to prepare smears. After Wright-Giemsa staining, bone marrow cellularity and hematopoietic activity were examined under Leica DM4000B inverted microscope (Leica, Wetzlar, Germany).

### Hematoxylin and eosin (H&E) staining

Thymus samples were fixed in 4% paraformaldehyde and subsequently embedded in paraffin. The paraffin-embedded tissues were cut into 4 µm slices and routinely stained with H&E for histopathological analysis. Random fields of each section were imaged under an inverted microscope (DMI4000B, Leica, Solms, Germany) at a magnification of 100 × and 400 × .

### Detection of leukocyte subpopulations in the spleen

Preparation of a single-cell suspension, a total of 3 × 10^6^ splenocytes were collected and incubated with Fixable Viability Stain 700 at room temperature. Subsequently, antibodies including APC-Cy7 anti-CD45, PE-CF594 anti-CD3, BV786 anti-CD45R/B220, BV510 anti-CD8, BV605 anti-F4/80, BV421 anti-Ly-6C, FITC anti-CD11b, and BV650 anti-Ly-6G were added at 1 µg each and incubated at room temperature for subsequent flow cytometric analysis (FACSCelesta, BD, MA, USA).

### Modulation of Th1, Th2, Th17, and Treg cells in the spleen

Splenocytes (2 × 10^6^ per well) were seeded in a 24-well plate and stimulated with Leukocyte Activation Cocktail for 5 h at 37 °C. Cells were then stained with Fixable Viability Stain 700 for 10 min in the dark at room temperature. Surface markers were labeled using PerCP-Cy5.5 anti-CD3 and BV650 anti-CD8, or BV421 anti-CD4, BB515 anti-CD25, and BV786 anti-KLRG-1, followed by fixation/permeabilization. Intracellular staining was performed using BV510 anti-IFN-γ, PE anti-IL-4, Alexa Fluor 647 anti-IL-17, or PE anti-Foxp3 antibody at 4 °C prior to analysis via flow cytometry (FACSCelesta, BD, MA, USA).

### Identification of Tn, Tem /Teff, and Tcm

A total of 2 × 10^6^ splenocytes were collected and stained with Fixable Viability Stain 700. Following this, the cells were incubated with PE-CF594-conjugated anti-CD3, BV510-conjugated anti-CD8, PerCP-Cy5.5-conjugated anti-CD44, and PE-conjugated anti-CD62L antibodies at room temperature in the dark. The samples were then prepared for flow cytometric analysis (FACSCelesta, BD, MA, USA).

### Detection of activation of T lymphocytes

On the day prior to the experiment, 48-well plates were coated with anti-CD3 antibody (2 μg/mL). Lymph node cells were then seeded at 1 × 10⁶ cells/mL and stimulated with recombinant IL-2 (10 ng/mL) and anti-CD28 antibody (1 μg/mL). Cell status and activation were assessed at 36 and 72 h, and clusters ≥ 50 μm in diameter were considered clones. Clone sizes were measured using Image J. Cells were subsequently stained with Fixable Viability Stain 700 and antibodies against CD4, CD8, CD25, and CD27 for flow cytometric analysis (FACSCelesta, BD, MA, USA).

### Detection of T cell proliferation

In a 96-well plate coated with CD3 at a concentration of 2 µg/mL, lymphocytes were labeled with CFSE at a concentration of 1 × 10⁶ cells/mL and incubated for 72 h. At 72 h post-seeding, the cells were collected and stained with BV605 anti-CD4 and BV510 anti-CD8 antibodies, then incubated at room temperature in the dark for subsequent flow cytometric analysis (FACSCelesta, BD, MA, USA).

### Detection of perforin and IFN-γ in CTLs

Following 36 h of in vitro stimulation, lymph node cells were treated with Leukocyte Activation Cocktail for the final 4 h. After collection and washing, cells were stained with Fixable Viability Stain 700, followed by surface staining with BV510 anti-CD8 antibody. Subsequent fixation/permeabilization was performed, and intracellular staining was carried out using APC-Perforin or PE-IFN-γ antibodies prior to flow cytometry analysis (FACSCelesta, BD, MA, USA).

### Mito-Tracker Deep Red 633 staining

Mito-Tracker Deep Red 633 is a membrane potential-dependent mitochondrial probe, exhibiting stronger red fluorescence in mitochondria with higher membrane potential. Upon depolarization, it disperses into the cytoplasm and fluorescence diminishes. After 48 h of stimulation, lymphocytes were stained with pre-warmed Mito-Tracker Deep Red 633 at 37 °C for 25 min in the dark. Following buffer addition, cells were imaged by Leica SP5 confocal laser scanning microscope (Leica, Wetzlar, Germany). The percentage of red fluorescent cells was calculated as (red fluorescent cells / total cells) × 100%.

### JC-1 assay for mitochondrial membrane potential

A total of 1 × 10^6^ lymphocytes stimulated in vitro for 48 h were collected and washed with PBS. The cells were then resuspended in JC-1 working solution and incubated at 37 °C with 5% CO₂ for 15–20 min. After incubation, the cells were centrifuged at 2000 rpm for 5 min, collected, and washed twice with 1 × Incubation Buffer. Finally, the cells were resuspended in 1 × Incubation Buffer and subjected to flow cytometry analysis (FACSCelesta, BD, MA, USA).

### Seahorse metabolic assay

The sensor cartridge was hydrated overnight. Cells were resuspended in pre-warmed assay medium (1 × 10^5^ cells/well) and seeded at 50 µL/well, with assay medium alone added to background wells. After centrifugation at 200 × g for 1 min, the plate was incubated at 37 °C without CO₂ for 25–30 min to facilitate adhesion. Pre-warmed medium was then carefully added along the well walls, followed by an additional 15–25 min of incubation.

For the mitochondrial stress test, sequentially inject oligomycin, Carbonyl cyanide 4-(trifluoromethoxy)phenylhydrazone (FCCP), and rotenone/antimycin A into ports A, B, and C, respectively. For glycolysis rate measurement, inject 2-DG and rotenone/antimycin A into ports A and B. For real-time glycolytic ATP rate assay, inject oligomycin and rotenone/antimycin A into ports A and B. After adding the reagents, initiate measurement on the Seahorse XFe96 Analyzer (Agilent Technologies, CA, USA).

### Establishment of A20 tumor-bearing mouse model and experimental grouping

6 specific-pathogen-free (SPF) grade BALB/c mice, male, 7 weeks old, 20–22 g, were purchased from SLAC Laboratory Animal Technology Co., Ltd. (Shanghai, China). All mice were kept in a clean facility with controlled temperature (22–26 °C) and humidity (30–50%). Experiments were approved by the Fujian University of Traditional Chinese Medicine Institutional Animal Care and Use Committee (approval No. FJTCMIACUC2023085).

Mice were randomly divided into two groups (n = 3): the DOX group (5 mg/kg/day via tail vein injection) and the DOX + QFQ group (DOX 5 mg/kg/day intravenously plus QFQ 4.5 g/kg/day orally). Both groups received DOX once every 5 days for a total of 3 doses. The DOX + QFQ group additionally received daily QFQ for 22 consecutive days. After treatment, 1 × 10⁶ A20 cells were subcutaneously inoculated into the right dorsal flank of each mouse. On day 21 post-inoculation, all mice were euthanized, tumors were excised and weighed, and single-cell suspensions (2 × 10⁶ cells) were prepared for staining with Fixable Viability Stain 700 and fluorescent antibodies against CD16/32, CD45, CD3, CD8, PD-1, CD28, and CTLA-4. The samples were then examined for flow cytometric analysis (FACSCelesta, BD, MA, USA).

### Statistical analysis

GraphPad Prism 10.0 was used to plot the data collected for this investigation, while SPSS 25.0 was used for analysis. The independent samples *t*-test was employed for comparisons between the two groups. One-way ANOVA was utilized for comparisons between several groups, whereas repeated measures ANOVA was utilized for continuous data across time. The mean ± standard deviation ($\overline{x }$± SD) is used to display the data. *P*-values below 0.05 were regarded as statistically significant. The mechanism map was generated using Figdraw software.

## Results

### Active ingredients of QFQ and their potential immune-related targets were analyzed through GO and KEGG pathway enrichment analysis.

**Fig. 1:**
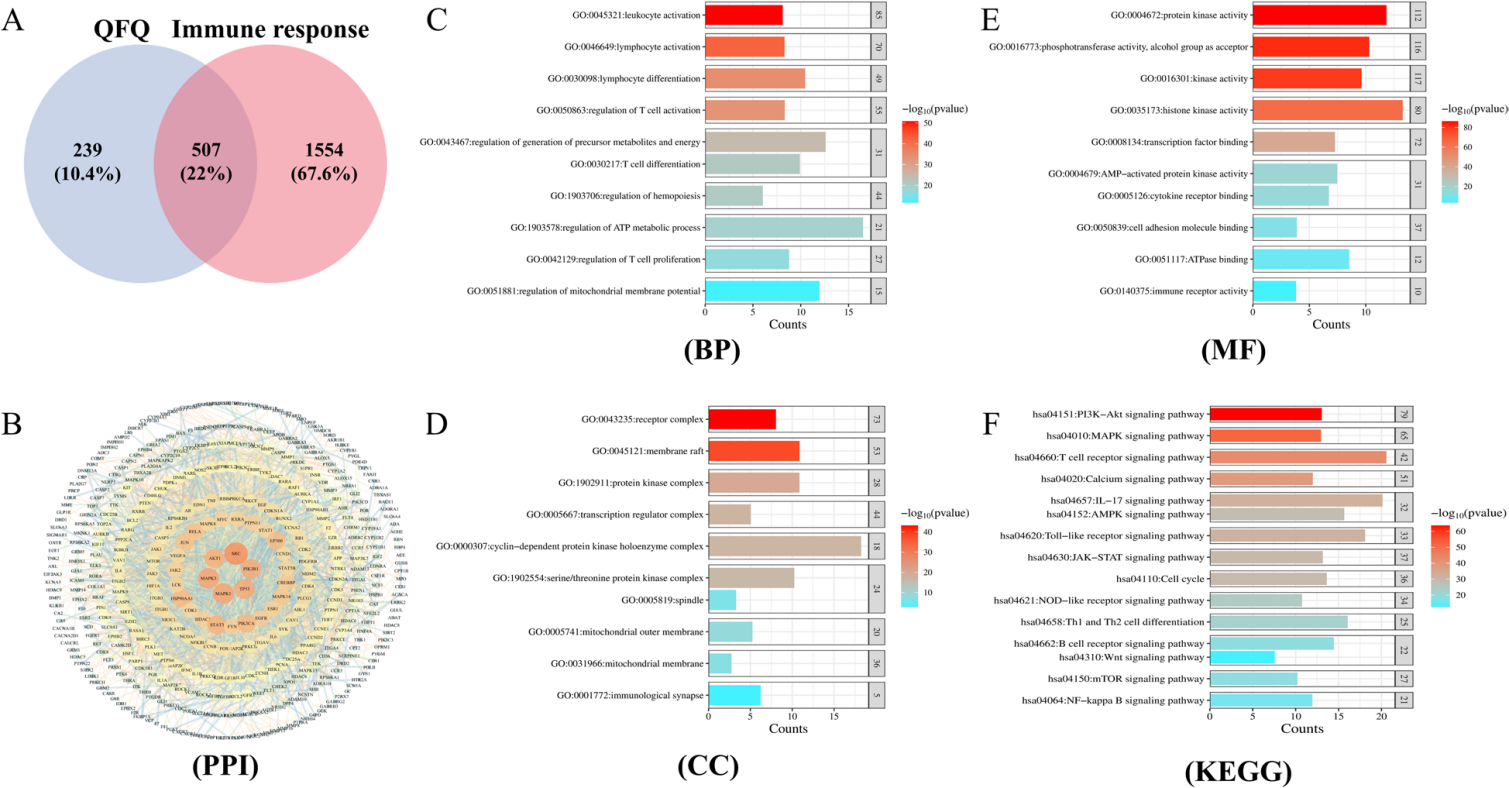
QFQ and immune response-related targets, PPI network in immune treatment and analysis of GO and KEGG enrichment. **A** Venn diagram of shared QFQ and colon immune response- related targets. **B** Visualization of the PPI network by Cytoscape 3.9.1. Using the Centiscape 2.2 plugin in Cytoscape 3.9.1, the lines within the PPI network were visually organized according to their degree values, reflecting the strength of protein interactions. Larger nodes with deeper colors and closer proximity to the center indicated higher degree values, signifying stronger interactions among the targets. Top 10 GO terms in **C** biological process (BP), **D** Cellular component (CC) and **E** Molecular function (MF) categories. **F** Top 15 KEGG pathways

A search of the TCMSP database revealed 130 bioactive components of QFQ, removing invalid and shared target ingredients yielded 57 ingredients (Supplemental Table 1). 746 QFQ projected targets in total were gathered. Additionally, a search with the keyword “Immune response” in the GeneCards database resulted in 2061 targets associated with immune system. Venny 2.1 software was used to identify the shared targets, resulting in 507 common targets (Fig. 1A), which could be potential targets for QFQ in treating immune system.

To analyze the interactions between QFQ compounds and immune-related targets, we utilized the STRING database to analyze 507 shared targets and construct a PPI network. The network was visualized using Cytoscape 3.9.1 (Fig. 1B). The top 20 degree values were chosen as the main targets in the PPI network: JUN, LCK, CDK1, TNF, FOS, IL-6, IL-2, MTOR, IL-1β and others (Table 1).

**Table 1:** Key target information of QFQ

Target	Degree	Betweenness unDir	Closeness unDir
SRC	100	21,878.05	0.00114
MAPK1	85	9059.28	0.00110
MAPK3	84	8689.13	0.00111
TP53	80	18,747.66	0.00109
PI3KR1	78	5446.37	0.00102
AKT1	75	10,740.29	0.00108
STAT3	73	6787.83	0.00107
JUN	59	5833.93	0.00106
LCK	46	1142.94	0.00097
MYC	45	2253.54	0.00101
CDK1	43	3599.61	0.00097
TNF	37	2693.48	0.00094
FOS	37	1929.50	0.00099
IL-6	33	1911.92	0.00091
HIF1α	33	1404.27	0.00098
IL-2	32	360.40	0.00093
CDK4	28	588.54	0.00092
MTOR	25	1102.61	0.00091
PTEN	23	2042.12	0.00089
IL-1β	22	1099.73	0.00091

GO functional analysis is a systematic methodology for annotating and performing enrichment analysis of gene or protein functions based on the Gene Ontology framework. It primarily interprets gene function through three core aspects: biological process (BP), cellular component (CC), and molecular function (MF). The top 10 enriched terms from each category were visualized in a bar chart. BP terms mainly involved leukocyte and lymphocyte activation and differentiation; regulation of precursor metabolites and energy production; T-cell activation and differentiation; regulation of ATP metabolic processes; T-cell proliferation; and regulation of the mitochondrial membrane potential and others (Fig. 1C). CC terms were related to receptor complexes, transcriptional regulatory complexes, cell cycle-dependent protein kinases, spindle apparatus, mitochondrial outer membrane and immunological synapses, etc. (Fig. 1D). MF terms were associated with Protein kinase activity; phosphotransferase activity; transcription factor activity; AMP-activated protein kinase activity; cell adhesion molecule activity; ATPase activity; immunoreceptor activity and others (Fig. 1E). These findings suggest that the functional status of T lymphocytes may be affected by QFQ through its multi-targeted immunomodulatory effects, which are closely related to mitochondrial function. Analysis of KEGG pathway enrichment (Fig. 1F) shows that among the pathways with the strongest gene correlations, the top 15 pathways included PI3K-Akt signaling pathway, T cell receptor pathway, Toll-like receptor pathway, mTOR signaling pathway, AMPK signaling pathway, NOD-like receptor pathway, Th1 and Th2 cell differentiation, etc. The core target genes of QFQ are closely related to the T-cell receptor signaling pathway and the Toll-like receptor pathway. The regulation of T-cell activation is significantly influenced by these mechanisms. Furthermore, QFQ participates in signaling pathways that are strongly linked to mitochondrial OXPHOS, glycolysis, and ATP metabolism, including PI3K-Akt, mTOR, and AMPK. This supports the findings of the GO functional enrichment study even further.

### QFQ improves the status of mice after DOX chemotherapy

**Fig. 2:**
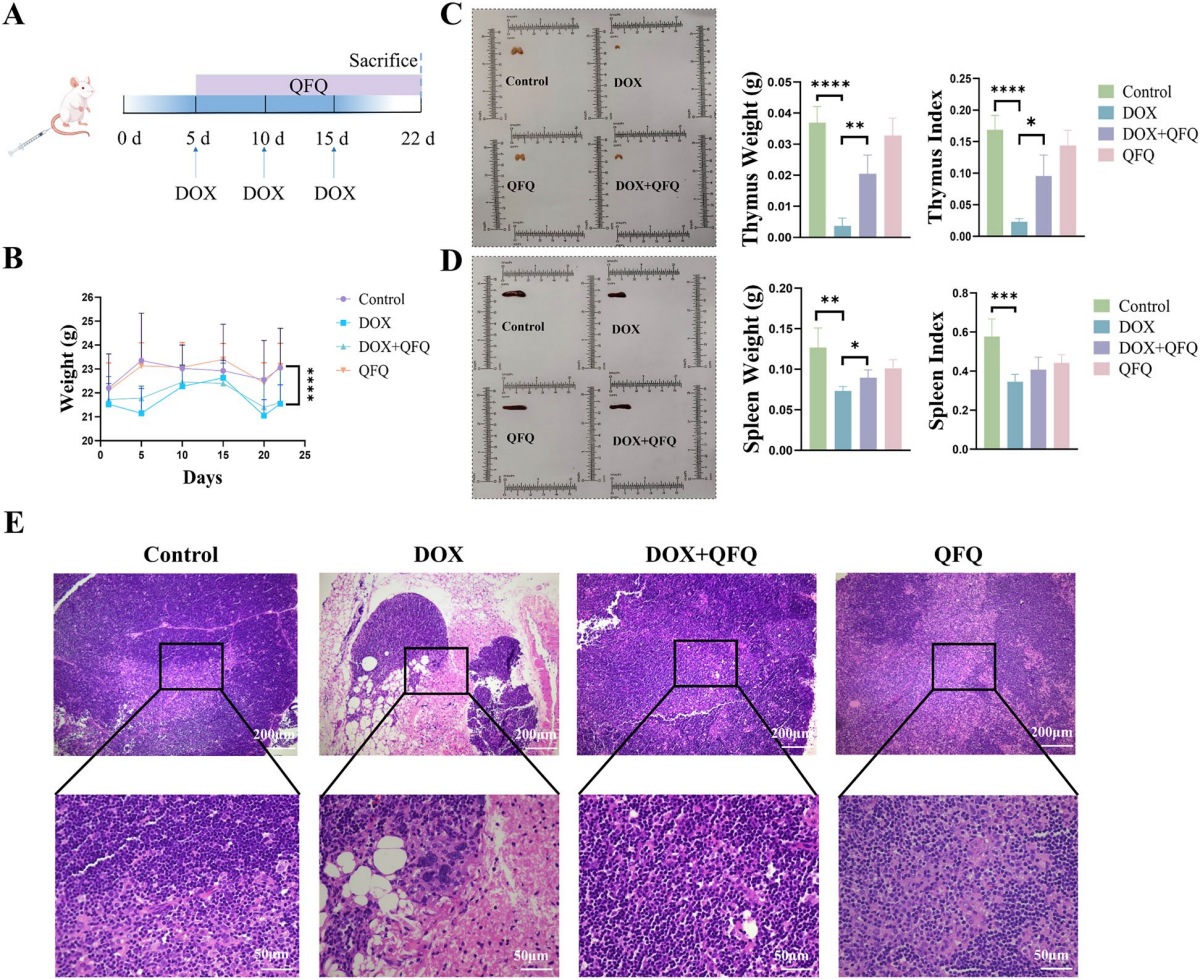
Effects of QFQ on body weight and immune organs in mice. **A** Schematic of the doxorubicin-induced mouse model and QFQ treatment regimen. **B** Effect of QFQ on body weight in mice. **C** Effect of QFQ on the weight and index of thymus in mice. **D** Effect of QFQ on the weight and index of spleen in mice. **E** Thymic tissue sections from each group of mice were analyzed by H&E staining. Representative H&E-stained images are shown (magnifications: 100 × , 400 ×). Data are presented as the mean ± SD, *P < 0.05, **P < 0.01, ***P < 0.001, ****P < 0.0001

The schematic diagram in Fig. 2A depicts the establishment of the DOX-induced immune dysregulation model in mice and the subsequent QFQ dosing regimen. QFQ by itself did not produce body weight loss, as Fig. 2B illustrates. QFQ demonstrated a protective impact on immunological organs, despite the fact that it did not considerably stop the weight loss brought on by DOX treatment. The DOX + QFQ group had significantly higher thymus weight (*P* < 0.01) and spleen weight (*P* < 0.05) as well as an enhanced thymus index (*P* < 0.05) when compared to the DOX group, as shown in Fig. 2C and D. These findings imply that by shielding immunological organs from the harm caused by chemotherapy, QFQ may mitigate DOX-induced immune dysregulation. Histological examination of thymic structure was done to further validate QFQ's protective impact on the thymus (Fig. 2E). The Control group's thymic lobules were well-defined, with identifiable corticomedullary boundaries and densely packed lymphocytes in the cortex. DOX therapy caused thymic structural disruption, which led to decreased lymphocyte counts, adipocyte-like vacuoles, and blurred corticomedullary borders. However, QFQ attenuates DOX-induced thymic damage, as evidenced by the well-defined thymic lobules with densely packed lymphocytes observed in the DOX + QFQ group.

### QFQ protects against DOX-induced bone marrow hematopoietic impairment

**Fig. 3:**
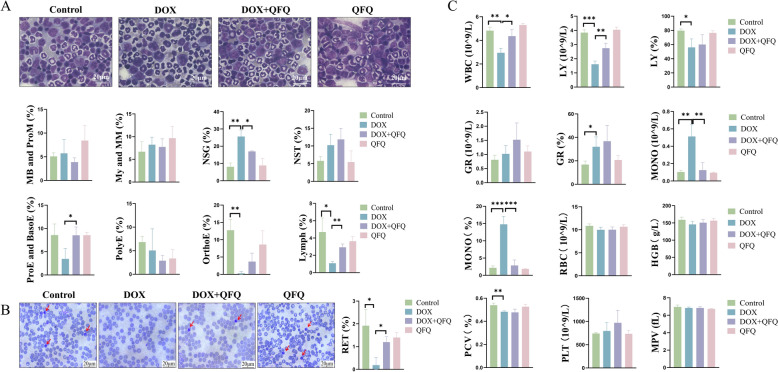
Effects of QFQ on bone marrow hematopoietic function in mice. **A** Representative bone marrow smears from each group of mice. MB and ProM: Myeloblast and Promyelocyte, My and MM: Myelocyte and Metamyelocyte, NSG: Neutrophil Segmented Granulocyte, NST: Neutrophilic Stab Cell, ProE and BasoE: Proerythroblast and Basophilic Erythroblast, PolyE: Polychromatic Erythroblast, OrthoE: Orthochromatic Erythroblast, Lymph: Lymphocyte. **B** Effect of QFQ on the proportion of reticulocytes in the peripheral blood of mice. Reticulocytes are indicated by red arrows. RET: Reticulocyte. **C** Effects of QFQ on hematological parameters in mice. WBC: White Blood Cell, Lymph: Lymphocyte, GR: Granulocyte, MONO: Monocyte, RBC: Red Blood Cell, HGB: Hemoglobin, PCV: Packed Cell Volume, PLT: Platelet, MPV: Mean Platelet Volume. Data are presented as the mean ± SD, *P < 0.05, **P < 0.01, ***P < 0.001

Bone marrow smears, reticulocyte counts, and complete blood count analyses were performed to evaluate the effects of QFQ on hematopoietic function in chemotherapy-treated mice. As can be seen in Fig. 3A, the DOX group had a large relative increase in segmented neutrophils (*P* < 0.01) and a significant decrease in the proportion of late erythroblasts (*P* < 0.01) and lymphocytes (*P* < 0.05) when compared to the Control group. This indicates that DOX significantly suppressed erythroid and lymphoid lineages, resulting in a relative increase in granulocytes. The DOX + QFQ group showed significantly increased proerythroblasts/early erythroblasts (*P* < 0.05), elevated lymphocytes (*P* < 0.01), and reduced segmented neutrophils (*P* < 0.05), demonstrating QFQ's protective effects on both erythroid and lymphoid lineages, which consequently led to a relative reduction in granulocytes.

Reticulocyte test results are shown in Fig. 3B. DOX significantly suppressed reticulocyte production (*P* < 0.05), consistent with the observed reduction of erythroid precursors in bone marrow (Fig. 3A). Notably, the DOX + QFQ group had a considerably greater reticulocyte proportion than the DOX group (*P* < 0.05), suggesting that QFQ partially restored erythropoietic function and mitigated the hematopoietic suppression brought on by DOX.

Routine blood tests also showed that the DOX group had significantly lower white blood cell (WBC) counts (*P* < 0.01), particularly lymphocyte counts (*P* < 0.001) and percentage (*P* < 0.05; Fig. 3C). The neutrophil percentage, on the other hand, rose (*P* < 0.05), as did the monocyte count (*P* < 0.01) and percentage (*P* < 0.001). The DOX + QFQ group showed a significant recovery in WBC count (*P* < 0.05) and lymphocyte number (*P* < 0.01) when compared to the DOX group. Furthermore, monocyte count and percentage dramatically decreased (*P* < 0.01/*P* < 0.001). Together, our findings imply that QFQ significantly reduces DOX-induced leukopenia, particularly by encouraging lymphocyte recovery. The conclusion that QFQ serves a protective role in maintaining bone marrow hematopoietic function after DOX treatment is supported by these data, which are in line with the bone marrow smear results.

### QFQ restores the proportion of T and B cells reduced by DOX in splenic leukocyte subpopulations

**Fig. 4:**
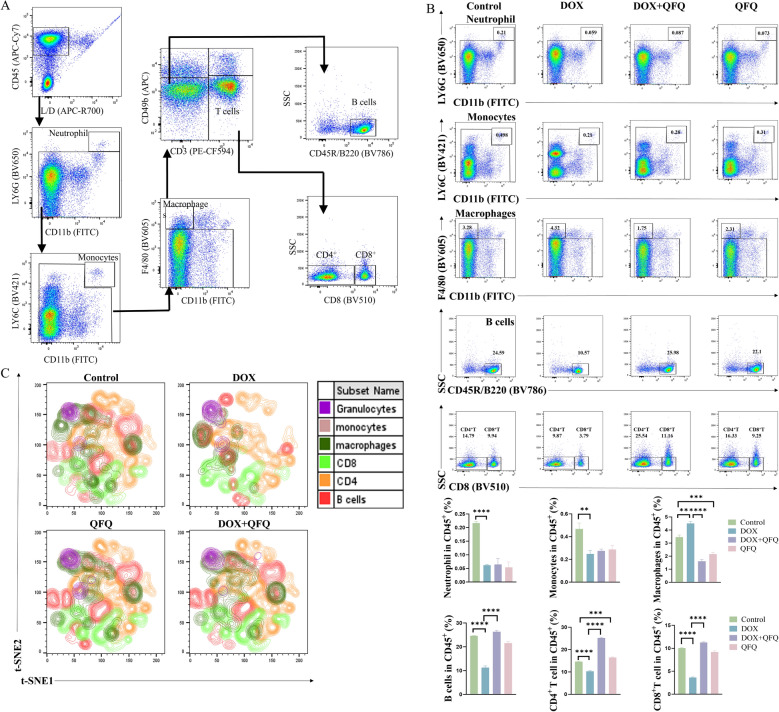
Effects of QFQ on the proportions of immune cell subsets in mouse splenocytes. **A** Flow cytometry gating strategy diagram. **B** The frequency of immune cell subsets among CD45⁺ leukocytes is shown in representative flow cytometry plots and quantified in the accompanying figures. **C** t-SNE analysis of immune cell subsets in mouse splenocytes. Data are presented as the mean ± SD, **P < 0.01, ***P < 0.001, ****P < 0.0001

Given that QFQ alleviates DOX-induced splenic atrophy and that the spleen serves as a critical site for T/B cell immune responses, we hypothesized that QFQ might modulate the proportions of splenic immune cells. To test this, we quantified the major leukocyte subsets in the spleen, with a focus on T and B lymphocytes. The gating strategy for flow cytometry populations is shown in Fig. 4A. As illustrated in Fig. 4B, the DOX group had considerably lower proportions of granulocytes, monocytes, CD4⁺T cells, CD8⁺T cells, and B cells (*P* < 0.01/*P* < 0.0001) than the Control group, whereas the proportion of macrophages was significantly higher (*P* < 0.01). The proportions of B cells, CD4⁺T cells, and CD8⁺T cells were significantly higher (*P* < 0.0001) in the DOX + QFQ group than in the DOX group, while the proportions of macrophages were significantly lower (*P* < 0.0001). To further visualize these differences, we conducted t-SNE dimensionality reduction analysis on leukocyte subpopulations between groups (Fig. 4C). This analysis clearly demonstrated a significant drop in total splenic leukocytes in the DOX group, predominantly due to fewer T and B cells. Importantly, both subsets were substantially restored following QFQ treatment. Taken together, these results demonstrate that DOX disrupts the composition of the splenic immune cells, leading to a significant reduction of T and B cell populations along with key innate subsets, accompanied by a compensatory increase in macrophages. Notably, QFQ seems to counteract these alterations by selectively restoring the frequencies of T and B cells and reducing the counts of macrophages. This process potentially rebalances the immune landscape and reestablishes conditions favorable for adaptive immunity following chemotherapy-induced damage. Furthermore, our study revealed that QFQ treatment alone increased the proportion of CD4⁺T cells, suggesting a potential role in enhancing Th cell-mediated cellular immunity. Additionally, t-SNE analysis indicated that DOX induced a more pronounced reduction in B cells compared to T cells. In contrast, QFQ specifically counteracted this B cell loss. The mechanism underlying this selective recovery of B cells will be a key focus of our future research.

### QFQ promotes Th1/Th2 differentiation, inhibits Treg suppressive receptor expression, and protects Tcm and Tem/Teff from DOX-induced damage.

**Fig. 5:**
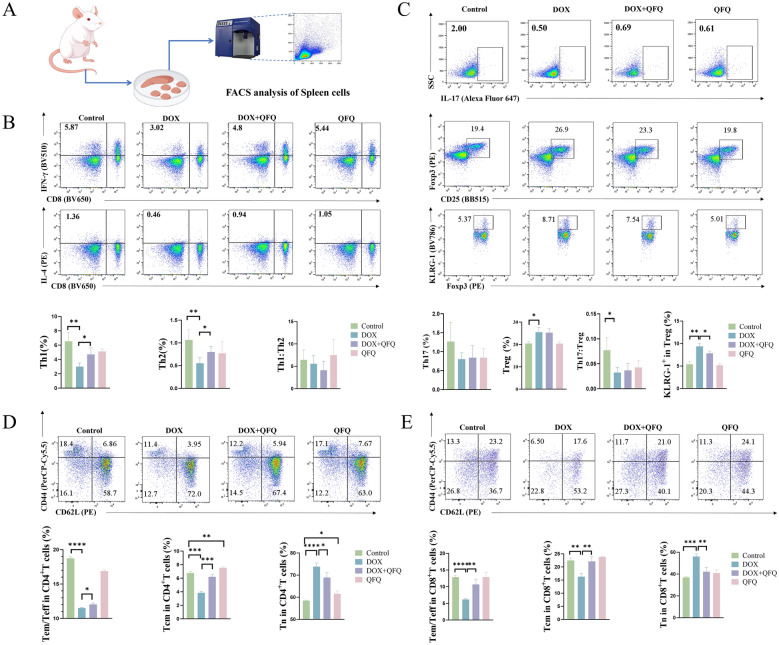
Effects of QFQ on splenic T cell subsets in mice. **A** Flow cytometry analysis of major immune cell populations in the spleen. **B** Frequency of Th1, Th2 cells and the Th1/Th2 ratio among CD3⁺T cells. **C** Frequency of Th17, Treg, KLRG-1⁺ Treg cells and the Th17/Treg ratio among CD3⁺T cells. **D** Composition of Tem/Teff, Tcm and Tn within CD4^+^T cells. **E** Composition of Tem/Teff, Tcm and Tn within CD8^+^T cells. Data are presented as the mean ± SD, *P < 0.05, **P < 0.01, ***P < 0.001, ****P < 0.0001

Figure 5A shows a schematic diagram of flow cytometric analysis of T cell subsets in the spleen of mice. As shown in Fig. 5B, DOX treatment significantly reduced the proportions of Th1 and Th2 cells within the CD4⁺T cell population (*P* < 0.01), although the Th1/Th2 ratio remained unchanged. Compared with the DOX group, the DOX + QFQ group showed a significant increase in both Th1 and Th2 cell proportions in the spleen (*P* < 0.05). As shown in Fig. 5C, DOX did not significantly affect the proportion of Th17 cells but significantly increased the proportion of Treg cells and their KLRG-1⁺ subset (*P* < 0.05/*P* < 0.01), resulting in a significantly decreased Th17/Treg ratio (*P* < 0.05). Compared with the DOX group, the DOX + QFQ group exhibited a significant reduction in the proportion of KLRG-1⁺ Treg cells (*P* < 0.05), although the Th17/Treg ratio was not markedly improved. These findings suggest that DOX selectively suppresses the differentiation of effector T cell subsets such as Th1 and Th2, while promoting the expansion and suppressive capacity of Treg cells, thus skewing the immune response toward a regulatory phenotype. QFQ may partially reverse DOX-induced adaptive immune dysfunction by restoring the differentiation of effector CD4⁺T cell subsets (Th1 and Th2) and reducing the expression of the inhibitory receptor KLRG-1 on Treg cells.

We further evaluated the distribution of Tn, Tcm, and Tem/Teff in the spleen using multicolor flow cytometry. As shown in Fig. 5D and E, CD44⁻CD62L⁺cells represent Tn, CD44⁺CD62L⁺cells represent Tcm, and CD44⁺CD62L⁻cells represent Tem/Teff. Compared with the Control group, QFQ affected the proportions of Tcm and Tn. In the DOX group, both CD4⁺and CD8⁺T cells showed a significant reduction in Tem/Teff proportions (*P* < 0.0001), a decrease in Tcm proportions (*P* < 0.001/*P* < 0.01), and an increase in the proportion of Tn (*P* < 0.0001/*P* < 0.001). Compared with the DOX group, the DOX + QFQ group exhibited significantly increased proportions of both Tem/Teff (*P* < 0.05/*P* < 0.01) and Tcm (*P* < 0.001/*P* < 0.01), along with a reduction in Tn (*P* < 0.05/*P* < 0.01). According to these findings, Tn, which are comparatively quiescent, are less impacted by DOX than rapidly proliferating cells, such as Tcm and Tem/Teff subsets. The naïve T cell population rises proportionately as a result of this change. In addition to successfully restoring the distribution and heterogeneity of T cell subsets necessary for a capable adaptive immune response, QFQ seems to specifically shield Tcm and Tem/Teff from DOX-induced cytotoxicity.

### *QFQ alleviates DOX-induced suppression of T cell activation *in vitro

**Fig. 6:**
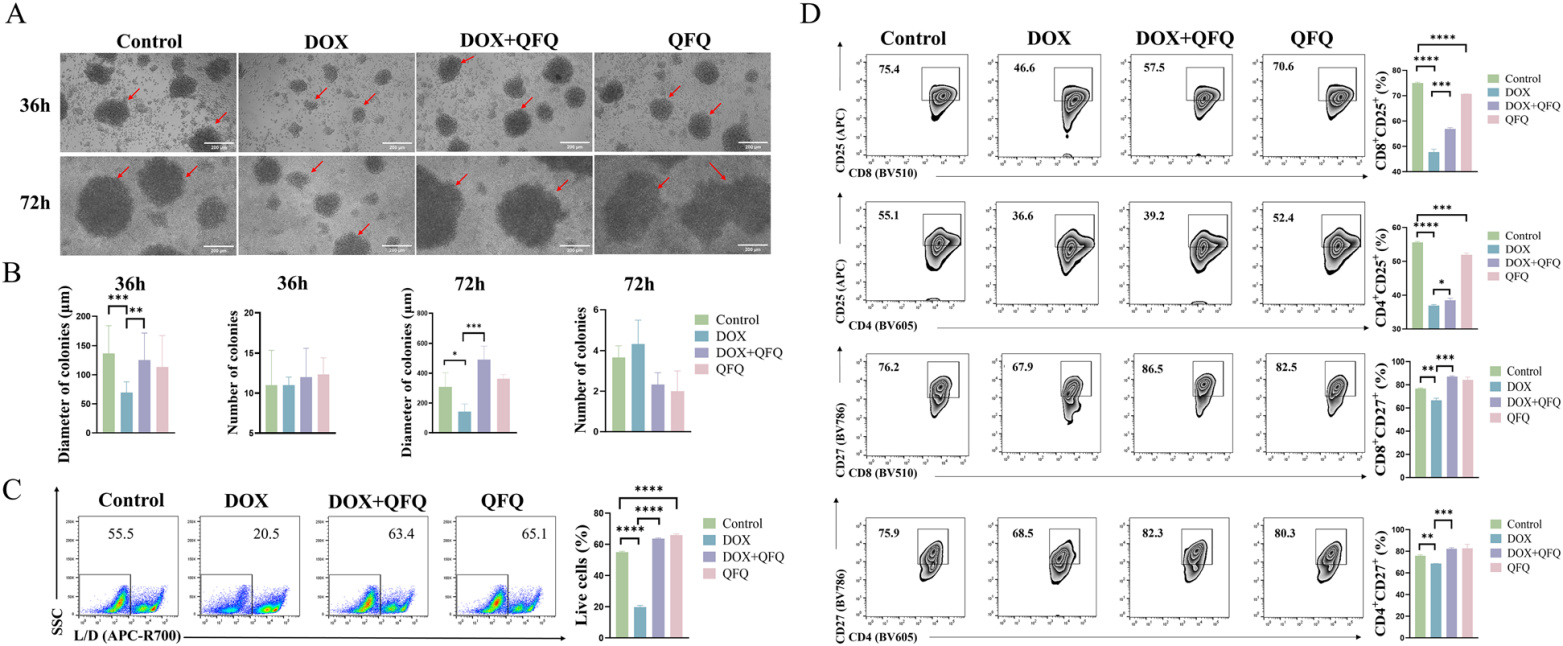
Effects of QFQ on T cell activation and viability in mice. **A** Representative images of cell activation at 36 h and 72 h, with cell clones indicated by red arrows. (magnifications: 100 ×). **B** Comparison of the number and diameter of cell clones among the groups. **C** Effects of QFQ on cell viability in mice. **D** Flow cytometry analysis of the expression of CD25⁺ and CD27⁺ among CD4⁺ or CD8⁺T cells. Data are presented as the mean ± SD, *P < 0.05, **P < 0.01, ***P < 0.001, ****P < 0.0001

An in vitro T cell culture system was set up and monitored at 36 and 72 h, as seen in Fig. 6A. At 36 h, lymphocytes in the Control and QFQ groups showed diverse sizes and morphology, dense distribution, massive expansion, and the formation of clustered clone spheres, all typical signs of T cell activation. The DOX group's lymphocytes, on the other hand, showed less of the asymmetrical size and shape linked to activation and instead looked uniformly spherical. Additionally, certain cells had reduced refractivity, shrinkage, and fragmentation, suggesting that DOX treatment impairs cell survival and inhibits T cell activation. The DOX + QFQ group's lymphocytes were noticeably bigger, more morphologically varied, and more reactive than the DOX group. This suggests that QFQ improves cell survival and lessens the DOX-induced suppression of T cell activation. By 72 h, clonospheres had greatly expanded and cell clusters had grown denser as a result of widespread proliferation. The average diameter of clonospheres was significantly smaller in the DOX group (*P* < 0.05), suggesting that DOX inhibits T cell activation and proliferation, even though there was no significant difference in the number of clonospheres between the Control and DOX groups at 36 or 72 h, as seen in Fig. 6B. The DOX + QFQ group, on the other hand, had a considerable increase in clonosphere diameter (*P* < 0.01/*P* < 0.001), indicating that QFQ successfully restores the T cell proliferative potential that DOX has inhibited. We used flow cytometry to further evaluate lymphocyte viability in light of the enhancement in cell viability that QFQ was shown to provide under microscopy. As can be shown in Fig. 6C, the DOX group had a significantly lower cell survival rate (*P* < 0.0001) than the Control group. However, the DOX + QFQ group had a significantly higher survival rate (*P* < 0.0001). Interestingly, QFQ by itself also showed a little increase in cell viability (*P* < 0.0001). According to these results, QFQ considerably increases cell viability after DOX treatment. Subsequently, we evaluated the expression of CD25, a marker of T cell activation upon initial antigen stimulation, and CD27, a costimulatory molecule involved in T cell activation, function, and the magnitude of the immune response. As shown in Fig. 6D, following in vitro stimulation, the proportions of CD4⁺CD25⁺, CD8⁺CD25⁺, CD4⁺CD27⁺, and CD8⁺CD27⁺T cells were significantly reduced in the DOX group compared to the Control group (*P*<0.01/*P* < 0.0001). In contrast, these proportions were significantly increased in the DOX + QFQ group (*P* < 0.05/*P* < 0.001), indicating that DOX inhibits activation of both CD4⁺and CD8⁺T cells, while QFQ reverses this suppression. Furthermore, compared to the Control group, we found that QFQ administration alone decreased the expression of CD4⁺CD25⁺and CD8⁺CD25⁺, indicating that QFQ may have an impact on T cell activation when given alone.

### QFQ restores T cell proliferation and enhances CTL cytotoxic function following DOX chemotherapy

**Fig. 7:**
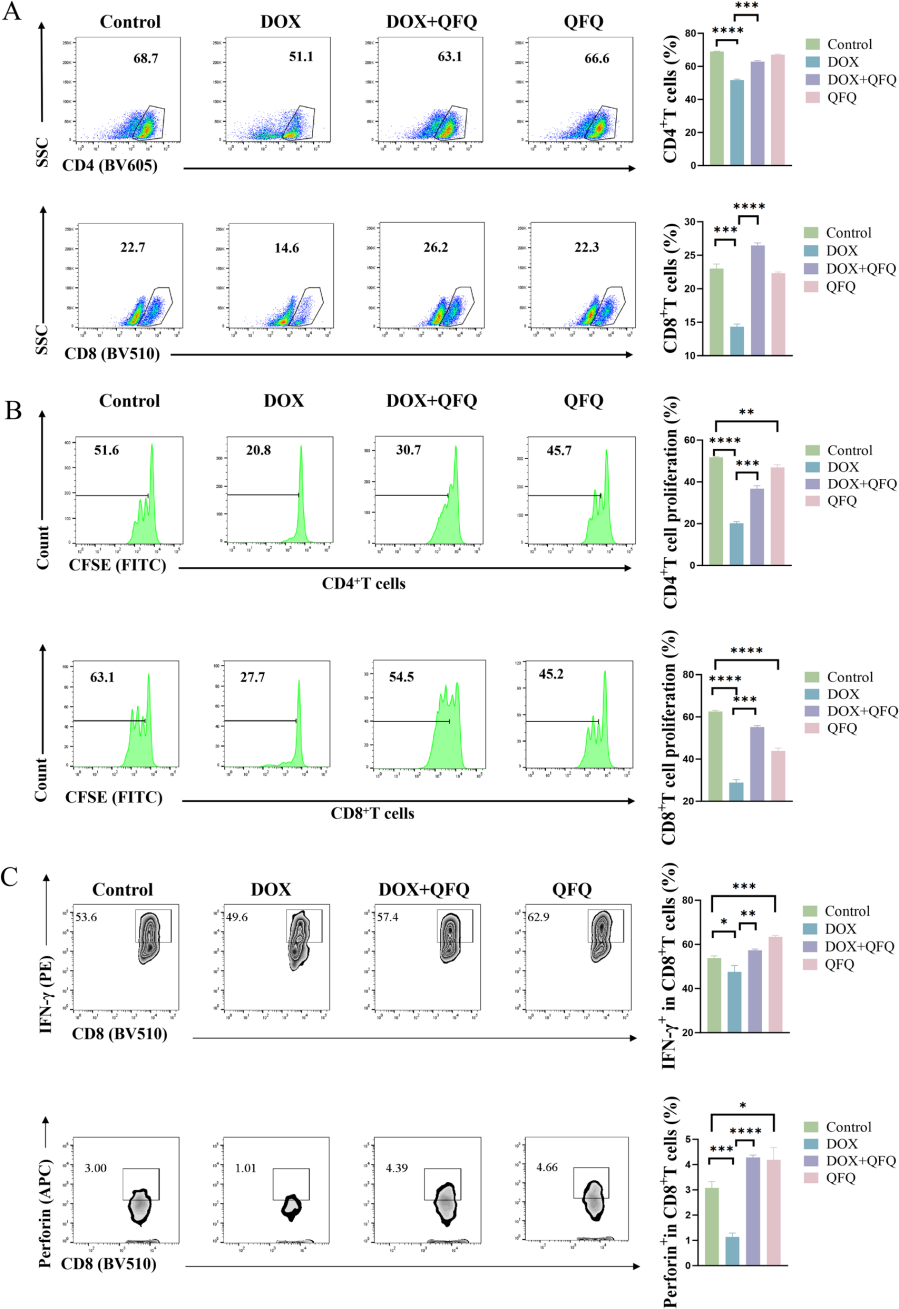
Profiling of T cell proliferation and CD8⁺T cell cytotoxic function. **A** Representative flow cytometry plots and statistical analysis of CD4⁺ and CD8⁺T cell proportions. **B** Proliferative capacity of CD4⁺ and CD8⁺T cells in vitro. **C** IFN-γ production and perforin expression in CD8⁺T cells. Data are presented as the mean ± SD, *P < 0.05, **P < 0.01, ***P < 0.001, ****P < 0.0001

The proportions of CD4⁺and CD8⁺T cells in the DOX group were considerably lower than those in the Control group following 72 h of in vitro stimulation and activation (*P*<0.001, *P*<0.0001), as seen in Fig. 7A and B. In contrast to the DOX group, QFQ therapy significantly raised the proportions of both CD4⁺and CD8⁺T cells (*P*<0.001/*P*<0.0001). CFSE proliferation assay demonstrated that in CD4⁺T cells, approximately 50% of lymph node cells in the Control and QFQ groups underwent cell division, whereas in the DOX group, T cell proliferation was markedly inhibited, with dividing cells accounting for only about 20% of the total population (*P* < 0.0001). Compared to the DOX group, QFQ significantly alleviated the division arrest (*P* < 0.001). A similar trend was observed in CD8⁺T cells. Compared to the Control group, the percentage of dividing cells in the DOX group was significantly decreased (*P* < 0.0001). In contrast, the DOX + QFQ group showed a significant increase in division index and proliferating cell proportion (*P* < 0.001), indicating that QFQ effectively counteracts DOX-induced inhibition of T cell proliferation and restores proliferative capacity. Furthermore, compared with the Control group, the QFQ group showed a significant reduction in T cell proliferation, accompanied by slower cell division (*P* < 0.01/*P* < 0.0001). Following activation and proliferation, T cells can further differentiate into CTLs, which mediate immune responses by secreting IFN-γ and perforin. To assess the effect of QFQ on CTL function, we examined intracellular cytokine expression. As shown in Fig. 7C, QFQ alone significantly increased the proportion of CD8⁺T cells expressing IFN-γ and perforin compared to the Control group (*P* < 0.001/*P*< 0.05). In the DOX group, the proportions of IFN-γ⁺ and perforin⁺CD8⁺T cells were significantly reduced (*P* < 0.05/*P* < 0.001). Notably, co-treatment with QFQ significantly restored IFN-γ⁺ and perforin⁺CD8⁺T cell frequencies in the DOX + QFQ group (*P* < 0.01/*P* < 0.0001). These results indicate that QFQ can mitigate DOX-induced impairment of CD8⁺T cell cytotoxic function. Also, we found that QFQ alone decreased the proliferation of both CD4^+^and CD8^+^T cells in comparison to the Control group. This is in line with previous research that demonstrated QFQ alone can inhibit T cell activation and proliferation. Moreover, QFQ monotherapy enhanced CTLs' capacity for cytotoxicity, demonstrating a functional profile of low proliferation but high cytotoxicity during T cell regulation.

### QFQ ameliorates DOX-induced mitochondrial membrane potential disruption in T cells

**Fig. 8:**
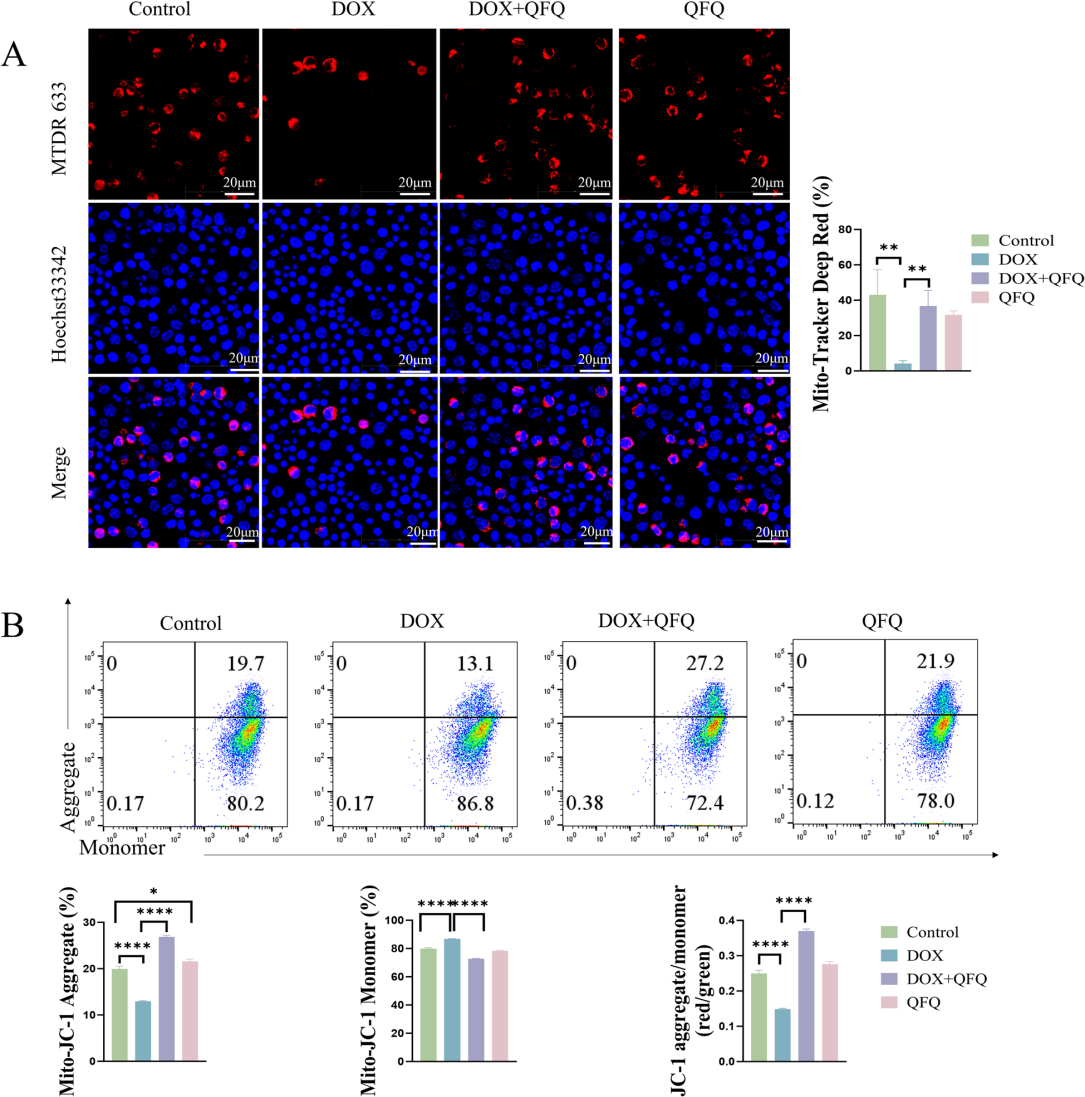
Effects of QFQ on mitochondrial membrane potential in mouse T cells. **A** Representative Mito-Tracker Deep Red 633 staining images and comparisons of red fluorescence expression levels among the groups. Mito-Tracker Deep Red 633 stains the mitochondria of live cells, emitting red fluorescence, while Hoechst 33,342 stains the nuclei, emitting blue fluorescence. MTDR 633: Mito-Tracker Deep Red 633. (magnifications: 1000 ×). **B** Representative JC-1 flow cytometry plots and quantitative analysis of red versus green fluorescence in each group. Data are presented as the mean ± SD, *P < 0.05, **P < 0.01, ****P < 0.0001

To explore whether QFQ protects T cells against DOX-induced damage by maintaining mitochondrial membrane potential (ΔΨm), we performed mitochondrial staining using Mito-Tracker Deep Red 633. A considerable decrease in membrane potential is anticipated since T cell activation in vitro entails metabolic reprogramming caused by oxidative stress, as illustrated in Fig. 8A. Compared to the Control group, the proportion of red-fluorescence-positive T lymphocytes was significantly reduced in the DOX group (*P* < 0.01), accounting for only 2–5% of total T cells, indicating DOX-induced mitochondrial dysfunction in T lymphocytes. In contrast, the DOX + QFQ group exhibited a marked increase in red-fluorescent T lymphocytes (approximately 20–40%, *P*<0.01), suggesting that QFQ may mitigate DOX-induced mitochondrial damage by preserving mitochondrial integrity or modulating oxidative stress pathways.

To further quantify the effect of QFQ on ΔΨm, we conducted JC-1 staining followed by flow cytometric analysis. In normal cells, JC-1 forms mitochondrial aggregates emitting red fluorescence, indicating intact function; in apoptotic cells, it remains as cytoplasmic monomers emitting green fluorescence, indicating dysfunction and apoptosis. As shown in Fig. 8B, compared to the Control group, T cells in the DOX group exhibited significantly reduced red fluorescence (*P* < 0.0001) and increased green fluorescence (*P* < 0.0001), resulting in a markedly decreased red/green fluorescence ratio (*P* < 0.0001). Notably, the DOX + QFQ group showed significantly enhanced red fluorescence (*P* < 0.0001), reduced green fluorescence (*P* < 0.0001), and a significantly increased red/green ratio (*P* < 0.0001) compared to the DOX group. These findings indicate that QFQ effectively alleviates DOX-induced mitochondrial membrane potential disruption, thereby improving the functional state of T lymphocytes.

### QFQ improves DOX-induced impairment of mitochondrial oxidative respiration and glycolytic function

**Fig. 9:**
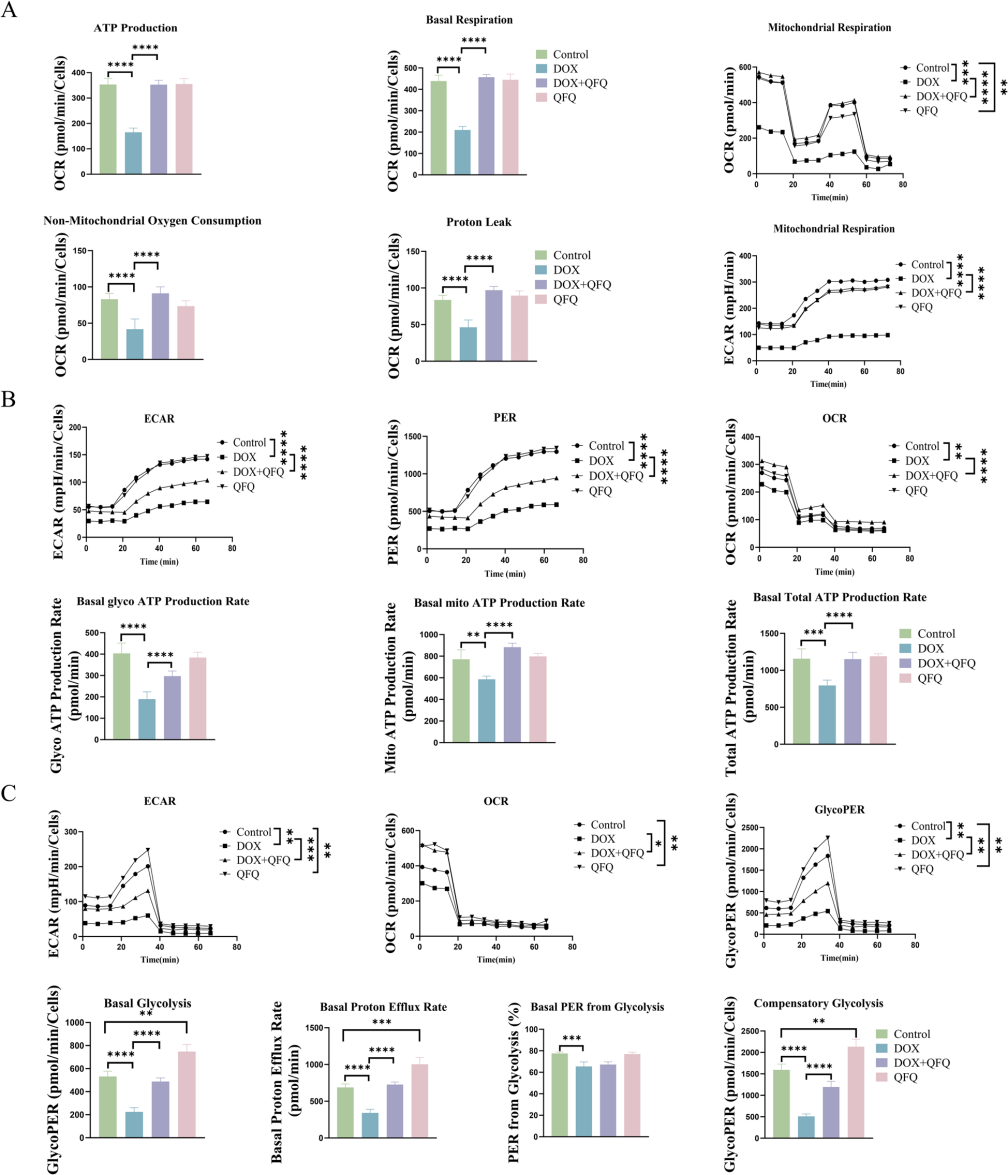
Effects of QFQ on cellular mitochondrial and glycolytic metabolism. Metabolic profiles were determined using Seahorse XF technology. **A** Comparisons of ATP production, basal respiration, OCR, non-mitochondrial oxygen consumption, proton leak, and ECAR among the groups in the mitochondrial stress test. **B** Comparisons of ECAR, PER, OCR, real-time glycolytic ATP production rate, real-time OXPHOS ATP production rate, and total ATP production rate among the groups in the real-time ATP rate assay. **C** Comparisons of ECAR, OCR, PER, basal glycolytic capacity, basal proton efflux rate, basal PER percentage from glycolysis, and compensatory glycolytic rate among the groups in the glycolytic function assay, *P < 0.05, **P < 0.01, ***P < 0.001, ****P < 0.0001

The mitochondrial stress test evaluates mitochondrial health and OXPHOS capacity by quantifying key OCR (Oxygen consumption rate, OCR) parameters: ATP production (oxygen used for ATP synthesis), representing energy output; basal respiration, indicating cellular energy demand; and proton leak, suggesting damage. Accurate measurement requires excluding non-mitochondrial oxygen consumption. Concurrent ECAR (Extracellular acidification rate, ECAR) monitoring reveals compensatory glycolysis activation upon mitochondrial suppression. Mitochondrial stress test results (Fig. 9A) showed that T cells in the DOX group exhibited impaired mitochondrial oxidative phosphorylation capacity, as evidenced by a reduction in ATP production, decreased basal respiration, significantly lower non-mitochondrial oxygen consumption, proton leak, ECAR (*P* < 0.0001), and OCR (*P* < 0.001), indicating that DOX severely disrupts mitochondrial aerobic respiration. In contrast, QFQ treatment in the DOX + QFQ group restored ATP production capacity, improved basal respiration, non-mitochondrial oxygen consumption, proton leak, and significantly increased ECAR and OCR values (*P* < 0.0001). These findings suggest that QFQ can ameliorate DOX-induced mitochondrial dysfunction and enhance mitochondrial energy metabolism.

To further investigate the temporal profile of ATP production through oxidative phosphorylation and glycolysis, we carried out a real-time ATP rate assay (Fig. 9B). OCR and ECAR are integrated into a mathematical model that ultimately outputs two quantitative ATP production rates. Rot/AA inhibition of mitochondrial respiration measures acidification, which with PER (Proton efflux rate, PER) computes glycolytic ATP production. Real-time glycolytic ATP production comes from glucose-to-lactate conversion; OXPHOS ATP is mitochondrial-derived. Total ATP equals their sum. Compared with the Control group, the DOX group showed significantly reduced ECAR, PER, and OCR (*P* < 0.01/*P* < 0.0001), along with decreased glycolytic ATP production rate (*P* < 0.0001), OXPHOS-associated ATP production rate (*P* < 0.01), and total ATP production rate (*P* < 0.001). Notably, QFQ treatment significantly restored ECAR, PER, OCR, ATP production via glycolysis, ATP production via OXPHOS and total ATP production (*P* < 0.0001), indicating that QFQ can improve both glycolytic and mitochondrial ATP production rates following DOX chemotherapy.

We will now begin the glycolytic capacity assay. Herein, the ECAR serves as a proxy for anaerobic metabolism and glycolytic flux, whereas the OCR is a measure of aerobic metabolic capacity. The PER provides insight into the overall metabolic balance and the cellular preference for OXPHOS versus glycolysis. Basal glycolysis measures glucose-to-lactate conversion. Basal proton efflux rate indicates glycolytic dependence, especially during mitochondrial impairment or hypoxia. Basal glycolytic PER percentage quantifies glycolysis-derived proton efflux. Compensatory glycolysis is the glycolytic rate after mitochondrial inhibition. Glycolysis rate analysis (Fig. 9C) revealed that compared to the Control group, the DOX group displayed decreased ECAR, glycolytic PER (*P* < 0.01), basal glycolytic capacity, basal proton efflux rate, percentage of glycolytic basal PER, and compensatory glycolysis (*P* < 0.001/*P* < 0.0001). Compared to the DOX group, the DOX + QFQ group showed significant improvements in ECAR, OCR, and glycolytic PER (*P* < 0.05/*P* < 0.01/*P* < 0.001), as well as enhanced basal glycolytic capacity, proton efflux rate, and compensatory glycolysis (*P* < 0.0001), suggesting that QFQ can restore glycolytic metabolism impaired by DOX. Moreover, compared with the Control group, QFQ alone also elevated glycolytic rates (*P* < 0.01/*P* < 0.001). All of these findings show that QFQ improves mitochondrial oxidative phosphorylation and glycolysis, which restores T cell metabolic capability. This subsequently promotes T cell activation and aids in immunological homeostasis regulation.

### QFQ enhances the anti-tumor effect and reverses T cell exhaustion in a post-chemotherapy recurrence model

**Fig. 10:**
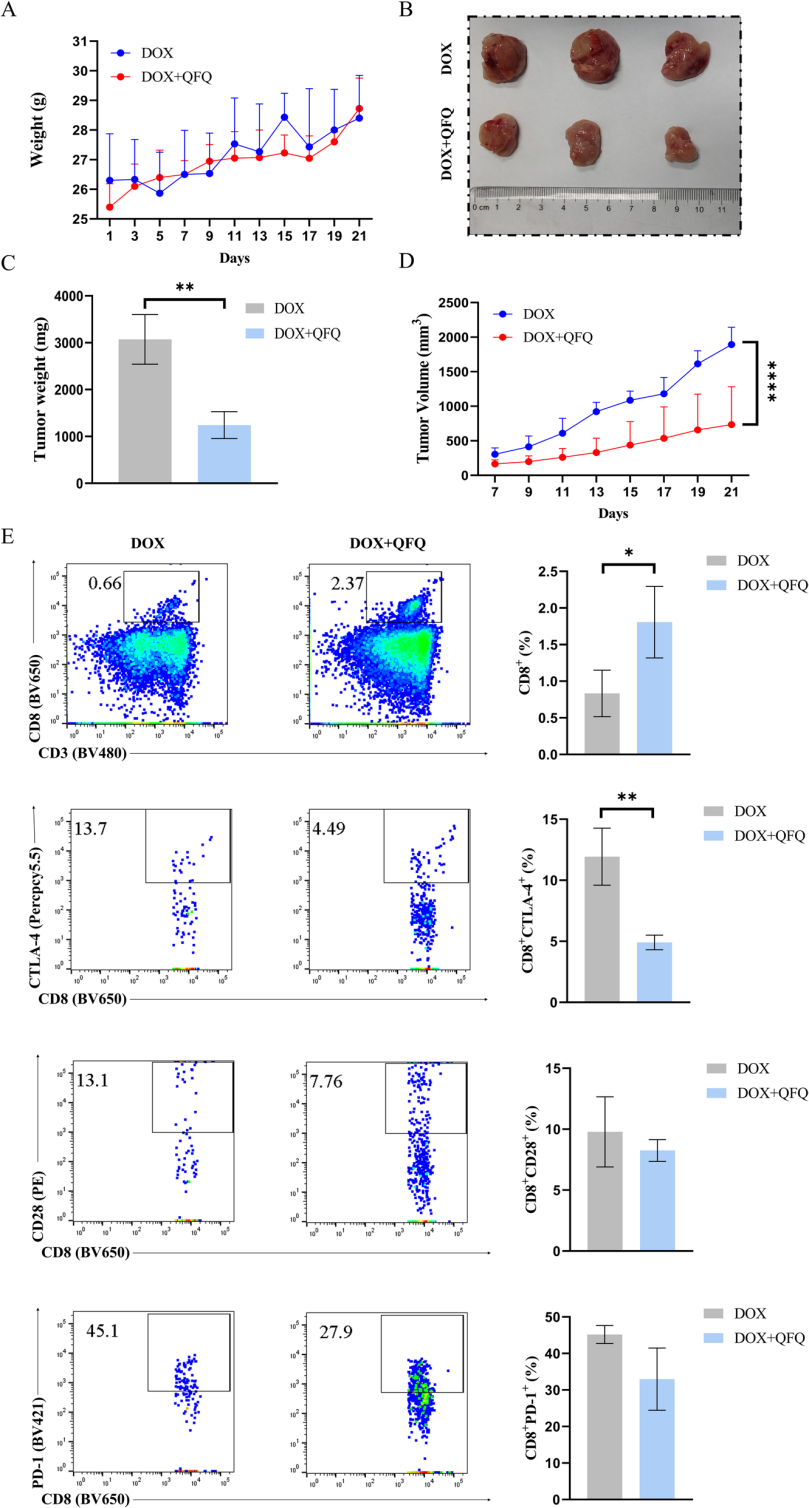
Anti-tumor activity of QFQ and its impact on CD8⁺T cell exhaustion in A20 tumor-bearing model. The therapeutic effects of QFQ were evaluated by monitoring body weight changes (**A**), visualizing tumor sizes (**B**), and quantifying tumor weight (**C**) and volume (**D**). The effects of QFQ on CD8⁺T cell exhaustion were investigated by analyzing CD8⁺T cell subsets, including PD-1, CTLA-4 and CD28 molecules (**E**), *P < 0.05, **P < 0.01, ****P < 0.0001

In the simulated post-chemotherapy tumor recurrence model, QFQ did not affect body weight (Fig. 10A) but significantly inhibited tumor growth. The DOX + QFQ group showed reduced tumor size, weight (*P* < 0.01; Fig. 10B and C) and volume (*P* < 0.0001; Fig. 10D) compared to DOX group, indicating potential antitumor effects of QFQ. Changes in tumor-infiltrating T lymphocytes were then analyzed using flow cytometry, and as Fig. 10E illustrates, the DOX + QFQ group had a higher proportion of CD8^+^T cells (*P* < 0.05) and a lower proportion of CD8^+^CTLA-4^+^T cells (*P* < 0.01). These findings suggest that QFQ may improve the anti-tumor activity of effector T cells, increase the tumor infiltration capacity of CD8^+^T cells, and downregulate CTLA-4 expression on CD8^+^T cells, thus reducing T cell exhaustion, improving the tumor immune microenvironment, and reversing the immunosuppressive effects caused by DOX.

## Discussion

QFQ has been widely utilized for decades during peri-chemotherapy for its multi-target, safe, and holistic benefits. Pharmacological studies show that Ganoderma lucidum and its polysaccharides promote immune organ growth, enhance macrophage activity, and increase IL-6 and TNF-α expression in immunosuppressed mice [26]. Astragalus polysaccharides, the primary active component of Astragalus membranaceus, activate dendritic cells, T and B lymphocytes, macrophages, NK cells, and microglia. They also promote the secretion of multiple cytokines and chemokines, thereby enhancing overall immune defense and surveillance [27]. Studies have demonstrated that Dioscorea opposita and its mucopolysaccharides increase IFN-γ production in murine splenocytes, activating cell-mediated immunity [28]. Ligustrum lucidum polysaccharides have the ability to increase NK cell activity, which strengthens immunological and anticancer responses [29]. Prunella vulgaris aqueous extract dose-dependently upregulates TNF-α, IL-1β, and IL-6 in RAW 264.7 cells. Hedyotis diffusa enhances effector and memory T cell responses and promotes tumor cell phagocytosis and clearance [30, 31]. Together, the combination of these six medicinal agents potentially possesses the ability to modulate the body's immune response and inhibit the growth of tumors.

In order to elucidate the mechanism by which QFQ modulates the body's immune response, we initially performed a network pharmacology analysis. This investigation suggested that QFQ may influence mitochondrial membrane potential and ATP metabolism pathways, while also modulating anti-tumor immunity and maintaining immune homeostasis through the T-cell receptor signaling pathway. Key monomeric components, including ganoderic Acid Mf, luteolin, quercetin, β-Sitosterol, kaempferol, and fomonitam, appear to be critical in QFQ's immunoregulatory effects. Notably, existing literature indicates that kaempferol has the ability to restore immunological homeostasis by modulating the Treg/Th17 balance through the miR-34a/Foxp3 axis [32]. By inhibiting the PD-1/PD-L1 pathway and reducing the synthesis of immunosuppressive factors by regulatory T cells, formononetin has been shown to activate CD8^+^T cells, hence having immunomodulatory and anticancer effects [33]. These findings have established a foundation for our subsequent research, which will focus on the mitochondrial metabolism pathway in T cells.

We created a DOX-induced mouse model of post-chemotherapy immune damage based on network pharmacology predictions. Our findings suggest that QFQ may help restore chemotherapy-impaired adaptive immunity because it prevented DOX-induced atrophy of the thymus and spleen, which are important organs for T cell generation and peripheral immunity, even if it did not significantly raise body weight. Analyses of peripheral blood and bone marrow showed that DOX significantly inhibited hematopoiesis, especially in late erythroblasts and lymphocytes, which resulted in a reduction in reticulocytes, lymphocytes, and hematocrit. Conversely, there was an observed increase in both the relative and absolute monocyte counts. A study has indicated that these alterations are predominantly attributable to chemotherapy-induced reactive myelopoiesis, which is skewed towards monocyte development. Furthermore, these changes have the potential to facilitate chemotherapy-induced tumor metastasis [34]. In the DOX + QFQ combination group, the observed decrease in both relative and absolute monocyte counts, along with a significant recovery in lymphocyte and reticulocyte proportions, suggests that QFQ effectively protects the bone marrow from DOX-induced damage and restores hematopoietic function, indicating a potential role in inhibiting chemotherapy-induced tumor metastasis.

To evaluate the protective effect of QFQ against DOX-induced impairment of adaptive immunity, we analyzed splenic leukocyte subpopulations. DOX reduced the numbers of B cells, neutrophils, monocytes, CD4⁺T, and CD8⁺T cells, while increasing the proportion of macrophages, consistent with previous findings [35, 36]. Following DOX chemotherapy, the administration of QFQ treatment has been observed to elevate the proportion of T cells and B cells in the spleen, with a particularly notable increase in B cell numbers. This effect is likely due to the active compounds present in QFQ, such as quercetin and luteolin. Quercetin has been shown to enhance the production of IgM-producing B cells and CD4⁺ and CD8⁺T cells [37, 38]. Luteolin inhibits solid tumor growth by sustaining CD8⁺T cell levels in the spleen, blood, and tumor tissue [39]. Given that the primary objective of this study is to investigate the anti-tumor immunity mediated by T cells as modulated by QFQ, the impact of QFQ on chemotherapy-induced B cells was not addressed in this research.

CD4⁺T cells differentiate into specialized subsets such as Th1, Th2, Th17, and Treg cells, which maintain immune balance through mutual cytokine regulation. However, altered cytokine profiles in the tumor microenvironment can disrupt this equilibrium, promoting a Th2-biased response that accelerates tumor development. The Th17/Treg balance plays a critical regulatory role in autoimmunity and tumorigenesis, sustaining immune tolerance while also contributing to tumor progression [40, 41]. This study revealed that DOX suppresses naïve T cell differentiation into Th1 and Th2 cells and reduces their splenic numbers, whereas QFQ preserves this differentiation capacity and mitigates CD4⁺T cell depletion. The KLRG-1⁺ Treg subset plays a key role in this process. As a co-inhibitory receptor highly expressed on senescent and exhausted NK and T cells, KLRG-1 suppresses immune activity by inhibiting AKT phosphorylation, thereby reducing proliferation and effector functions [42]. KLRG-1⁺ Treg cells are therefore thought to have a greater capacity for immunosuppression. KLRG-1⁺ Treg localization in tumors may have a more direct role in cancer cells' immune evasion [43]. Our findings showed elevated proportions of Treg cells and their KLRG-1⁺ subpopulation in the DOX group, accompanied by a distinct reduction in the Th17/Treg ratio. QFQ downregulated KLRG-1 in Tregs, reducing their suppression and alleviating immune dysregulation, suggesting a restored immune balance.

Based on antigen response, T cell subsets are characterized as Tn, Tem/Teff, and Tcm. Tn cells are activated and differentiate into effector or memory subsets upon exposure to antigens: Teff carry out direct immune responses, Tem offer quick cytokine-mediated defense. Upon re-exposure to antigen, Tcm proliferate rapidly and differentiate into Teff [44]. Our findings indicate that treatment with DOX resulted in an increase in CD4⁺Tn and CD8⁺Tn, while leading to a reduction in the proportions of Tem/Teff and Tcm. This is likely attributable to the greater chemosensitivity of proliferative and functional T cell subsets compared to the quiescent Tn cells, resulting in a relative increase in Tn cells. QFQ aids in restoring T cell diversity, thereby supporting adaptive immune responses.

We observed that QFQ significantly ameliorated DOX-induced alterations in leukocyte subsets, particularly the distribution of T lymphocyte subsets. However, it remained unknown whether QFQ could also improve T lymphocyte function. Therefore, we subsequently investigated the activation, proliferation, and cytotoxic function of T cells in mice following DOX chemotherapy and QFQ treatment. Morphological analysis at 36 and 72 h revealed that T cell clonal spheres in the DOX group were significantly smaller than controls. Both CD4⁺ and CD8⁺T cells exhibited reduced expression of CD25⁺ and CD27⁺, indicating impaired T cell activation and proliferation. In contrast, QFQ treatment increased clonal sphere size and elevated CD25⁺/CD27⁺ expression, suggesting that QFQ alleviates DOX-induced suppression of T cell activation. At 72 h after stimulation, the DOX group exhibited a significant decrease in the proportions of both CD4⁺ and CD8⁺T cells, according to CFSE labeling used to assess T-cell proliferation in the various treatment groups. Alongside this, there was a noticeable slowdown in T-cell division and a sharp decline in the number of proliferating cells throughout subsequent generations. On the other hand, QFQ successfully counteracted the DOX-induced inhibition of T-cell division and markedly raised the percentage of dividing cells.

After being activated and proliferating, T cells develop into CTLs, which prevent tumor development and metastasis by releasing cytotoxic granules such as granzymes and perforin. Our findings showed that DOX treatment dramatically decreased CTL production of IFN-γ and perforin. Nonetheless, QFQ medication successfully increased CTLs' ability to release these molecules, indicating a possible function for it in reversing DOX-impaired CTL cytotoxicity.

Taken together, these findings demonstrate that DOX disrupts the balance of T-cell subsets, suppresses the proliferation and differentiation of both CD4⁺ and CD8⁺T cells, diminishes antigen-specific activation, and impairs immunoregulatory and cytotoxic functions. QFQ counteracts these adverse effects by restoring T-cell subset balance, promoting T-cell proliferation and differentiation, reversing the suppression of T-cell activation, and ultimately ameliorating immune dysregulation.

Furthermore, it was observed that although QFQ alone diminished the activation and proliferation capacity of T cells in vitro, it concurrently enhanced the production of IFN-γ and perforin by CTLs. These results collectively suggest that QFQ skews T cell function toward a phenotype characterized by low proliferation but high cytotoxic potential, which aligns with previous findings. For example, β-sitosterol—a monomer compound in QFQ—has been shown to enhance CTL activity in tumor-bearing mice while suppressing CD4⁺CD25⁺ and CD8⁺CD25⁺ expression, inhibiting T cell activation, and reducing T cell proliferation [45, 46]. Likewise, it has been demonstrated that luteolin suppresses T cell activation by blocking CD25 and CD69 expression on CD4⁺T cells, while simultaneously increasing CTL cytotoxic activity and encouraging the release of IL-2, TNF-α, and IFN-γ [47, 48]. This "low proliferation, high cytotoxicity" phenomenon may be associated with oxidative stress during T-cell activation. QFQ, rich in antioxidants such as flavonoids and phytosterols, reduces excessive ROS production, thereby curbing overactivation and proliferation of T cells while preserving the effector function of CTLs. This moderated T-cell activation state not only ensures sustained cytotoxic activity but also aligns with the improved glycolytic capacity observed during T-cell proliferation and differentiation when QFQ is administered alone, collectively maintaining appropriate T-cell activation, effector function, and metabolic fitness.

Dynamic alterations in cellular metabolic processes are intimately associated with T cell lineage differentiation [49]. Under normal conditions, naive T lymphocytes rely primarily on OXPHOS to generate ATP via the TCA cycle and ETC for basic metabolic needs. However, as T cell activity rises, OXPHOS becomes insufficient to meet higher energy demands, prompting a shift to glycolytic pathways. Aerobic glycolysis not only supplies rapid ATP production but also suppresses OXPHOS, thereby limiting excessive ROS generation and protecting cells from oxidative damage [50]. This change, referred to as activation-induced metabolic reprogramming, occurs when activated effector T cells switch to aerobic glycolysis as their primary energy source, while naïve T cells continue to rely on OXPHOS [51]. Chemotherapeutic drugs can disrupt T cell energy homeostasis by impairing mitochondrial OXPHOS and inhibiting metabolic reprogramming through pathways such as PI3K-AKT-mTOR, AMPK, HIF-1α, and c-Myc. This disruption is a key mechanism underlying chemotherapy-induced T cell dysfunction. CD4⁺T cell subsets employ distinct metabolic programs upon differentiation. Th1 and Th17 cells preferentially utilize glycolysis, which enhances their secretion of IFN-γ and IL-17, respectively, to support anti-pathogen immunity [52]. Activated CD8⁺T cells differentiate into CTLs, whose cytolytic function depends on glycolysis. Enhanced glycolytic activity promotes the production of granzyme B, perforin, and IFN-γ, thereby improving CTL-mediated tumor clearance [53]. In conclusion, T cell activation, differentiation, and effector activities depend on both OXPHOS and glycolysis. Chemotherapy weakens immunological responses by destroying mitochondrial integrity, affecting ATP generation and biosynthesis, disrupting metabolic reprogramming, and preventing T cell proliferation. Chemotherapy has been linked in the past to mitochondrial malfunction, which includes decreased ATP production, structural abnormalities, and decreased mass [54]. Restoring mitochondrial energy metabolism, on the other hand, has been demonstrated to revitalize worn-out T cells, boosting their antiviral and anticancer immune responses [55]. The mitochondrial amount, membrane potential, and glycolytic ATP generation were evaluated in order to ascertain if QFQ mitigates DOX-induced T cell suppression through mitochondrial energy metabolism. The results demonstrated that DOX significantly decreased membrane potential and mitochondrial mass, which hindered ATP generation and the metabolic transition to aerobic glycolysis that is necessary for T cell activation. By restoring mitochondrial membrane potential and OXPHOS activity, QFQ promoted metabolic reprogramming and aided in T cell activation, proliferation, and differentiation.

In addition, this study investigated the potential role of QFQ in mitigating tumor recurrence under post-chemotherapy immunosuppressive conditions in mice. Using a post-chemotherapy tumor recurrence model, we found that mice treated with QFQ in combination with DOX showed significantly smaller tumor volumes compared to those treated with DOX alone, indicating that QFQ exerts unique therapeutic efficacy against chemotherapy-induced immunosuppression and thereby slows tumor progression. T cell exhaustion represents a core mechanism of tumor immune escape. By analyzing the key exhaustion marker CTLA-4 in tumor-infiltrating CD8⁺T cells, we demonstrated that QFQ effectively alleviates T cell exhaustion by downregulating CTLA-4 expression and enhancing CD8⁺T cell infiltration. These findings collectively indicate that QFQ enhances anti-tumor immunity by alleviating DOX-induced immune damage and remodeling the tumor immune microenvironment. Through immunological and metabolic system modulation, QFQ may act as a chemoadjuvant to increase efficacy and decrease toxicity.

## Conclusions

**Fig. 11:**
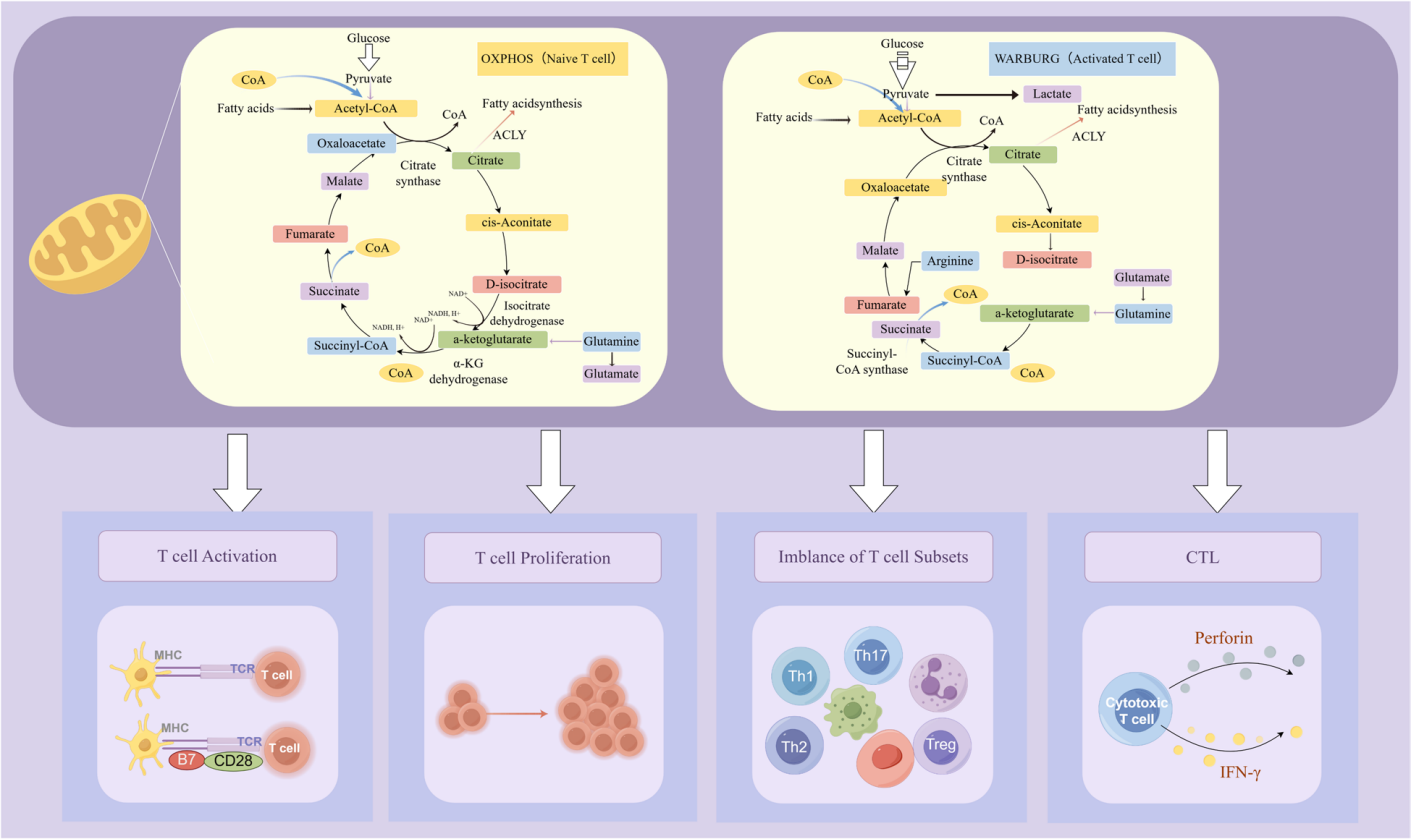
The potential mechanism by which QFQ regulates T cell immune dysfunction induced by DOX

QFQ may enhance mitochondrial membrane potential to restore T-cell metabolic capacity by regulating mitochondrial oxidative phosphorylation and glycolysis (Fig. 11). This metabolic repair restored CTL cytotoxicity, enhanced T-cell activation, and promoted proliferation. Thus, QFQ alleviates chemotherapy-induced immunodysregulation by rebalancing leukocyte subpopulations, improving bone marrow hematopoietic function after DOX chemotherapy, and improving the mice's general physiological status.

## Supplementary Information

Supplementary Material 1.

## Data Availability

No datasets were generated or analysed during the current study.

## Abbreviations

AMPK: AMP-Activated Protein Kinase
ATP: Adenosine triphosphate
BasoE: Basophilic erythroblast
CFSE: Carboxyfluorescein succinimidyl ester
CTL: Cytotoxic T lymphocyte
CTLA-4: Cytotoxic T-Lymphocyte-Associated Protein 4
DOX: Doxorubicin
ECAR: Extracellular acidification rate
ETC: Electron transport chain
GR: Granulocyte
HGB: Hemoglobin
HIF-1α: Hypoxia-inducible factor 1-α
IFN-γ: Interferon-γ
IL-2: Interleukin-2
KLRG-1: Killer cell lectin-like receptor G1
Lymph: Lymphocyte
MB: Myeloblast
MM: Metamyelocyte
MONO: Monocyte
MPV: Mean platelet volume
My: Myelocyte
NSG: Neutrophil segmented granulocyte
NST: Neutrophilic stab cell
OXPHOS: Oxidative phosphorylation
OrthoE: Orthochromatic erythroblast
OCR: Oxygen consumption rate
PCV: Packed cell volume
PER: Proton efflux rate
PLT: Platelet
PolyE: Polychromatic erythroblast
ProE: Proerythroblast
ProM: Promyelocyte
QFQ: Qiling Fuzheng Qingjie Granule
RBC: Red blood cell
RET: Reticulocyte
ROS: Reactive oxygen species
TCA: Tricarboxylic acid cycle
Teff: Effector T cell
Tem: Effector memory T cell
Tcm: Central memory T cell
Th1: T helper 1 cell
Th2: T helper 2 cell
Th17: T helper 17 cell
TNF-α: Tumor necrosis factor-α
Tn: Naïve T cell
Treg: Regulatory T cell
WBC: White blood cell
