# Supplementary material for: Mechanism of Qiling Fuzheng Qingjie granules in alleviating doxorubicin-induced T cell immune dysfunction via mitochondrial energy metabolism
Source: Chin Med. 2026 May 19;21:135. doi: 10.1186/s13020-026-01405-0 (PMC13185425; doi:10.1186/s13020-026-01405-0)
Supplement: Supplementary file 1 — Supplementary Material 1. [file 13020_2026_1405_MOESM1_ESM.docx]

**Supplemental table 1 Active Compound of QFQ**

| Molecular ID | Name | OB | DL | Source |  |
| --- | --- | --- | --- | --- | --- |
| MOL005212 | Olitoriside_qt | 103.23 | 0.78 | Ligustrum lucidum |  |
| MOL000546 | diosgenin | 80.88 | 0.81 | Dioscorea opposita |  |
| MOL000378 | 7-O-methylisomucronulatol | 74.69 | 0.3 | Astragalus membranaceus |  |
| MOL005190 | eriodictyol | 71.79 | 0.24 | Ligustrum lucidum |  |
| MOL000392 | formononetin | 69.67 | 0.21 | Astragalus membranaceus |  |
| MOL000433 | FA | 68.96 | 0.71 | Astragalus membranaceus |  |
| MOL000380 | (6aR,11aR)-9,10-dimethoxy-6a,11a-dihydro-6H-benzofurano[3,2-c]chromen-3-ol | 64.26 | 0.42 | Astragalus membranaceus |  |
| MOL001736 | (-)-taxifolin | 60.51 | 0.27 | Dioscorea opposita |  |
| MOL005430 | hancinone C | 59.05 | 0.39 | Dioscorea opposita |  |
| MOL004576 | taxifolin | 57.84 | 0.27 | Ligustrum lucidum |  |
| MOL006767 | Vulgaxanthin-I | 56.14 | 0.26 | Prunella vulgaris |  |
| MOL000211 | Mairin | 55.38 | 0.78 | Astragalus membranaceus |  |
| MOL000322 | Kadsurenone | 54.72 | 0.38 | Dioscorea opposita |  |
| MOL005147 | Lucidumoside D_qt | 54.41 | 0.47 | Ligustrum lucidum |  |
| MOL000371 | 3,9-di-O-methylnissolin | 53.74 | 0.48 | Astragalus membranaceus |  |
| MOL000239 | Jaranol | 50.83 | 0.29 | Astragalus membranaceus |  |
| MOL000354 | isorhamnetin | 49.6 | 0.31 | Astragalus membranaceus |  |
| MOL000439 | isomucronulatol-7,2'-di-  O-glucosiole | 49.28 | 0.62 | Astragalus membranaceus |  |
| MOL005146 | Lucidumoside D | 48.87 | 0.71 | Ligustrum lucidum |  |
| MOL011159 | ergosta-4,6,8(14),22-  tetraene-3-one | 48.32 | 0.75 | Ganoderma lucidum |  |
| MOL000417 | Calycosin | 47.75 | 0.24 | Astragalus membranaceus |  |
| MOL011168 | ergosta-7,9(11),22-trien-3β,  5α,6α-triol | 46.95 | 0.78 | Ganoderma lucidum |  |
| MOL000098 | quercetin | 46.43 | 0.28 | Hedyotis diffusa |  |
| MOL000737 | morin | 46.23 | 0.27 | Prunella vulgaris | |
| MOL005465 | AIDS180907 | 45.33 | 0.77 | Dioscorea opposita | |
| MOL001659 | Poriferasterol | 43.83 | 0.76 | Hedyotis diffusa | |
| MOL000449 | Stigmasterol | 43.83 | 0.76 | Hedyotis diffusa | |
| MOL006772 | Poriferasterol monoglucoside_qt | 43.83 | 0.76 | Prunella vulgaris | |
| MOL005440 | Isofucosterol | 43.78 | 0.76 | Dioscorea opposita | |
| MOL011137 | campesta-7,22E-dien-3beta-ol | 43.51 | 0.72 | Ganoderma lucidum | |
| MOL000282 | ergosta-7,22E-dien-3beta-ol | 43.51 | 0.72 | Ganoderma lucidum | |
| MOL011267 | Lucialdehyde B | 43.12 | 0.81 | Ganoderma lucidum | |
| MOL004355 | Spinasterol | 42.98 | 0.76 | Prunella vulgaris | |
| MOL011171 | Ganoderal B | 42.56 | 0.81 | Ganoderma lucidum | |
| MOL000422 | Kaempferol | 41.88 | 0.24 | Prunella vulgaris | |
| MOL004798 | Delphinidin | 40.63 | 0.28 | Prunella vulgaris | |
| MOL000442 | 1,7-Dihydroxy-3,9-dimethoxy pterocarpene | 39.05 | 0.48 | Astragalus membranaceus | |
| MOL011140 | 5alpha-Lanosta-7,9(11),24-triene-15alpha,26-dihydroxy-3-one | 38.54 | 0.81 | Ganoderma lucidum | |
| MOL000279 | Cerevisterol | 37.96 | 0.77 | Ganoderma lucidum | |
| MOL000953 | CLR | 37.87 | 0.68 | Dioscorea opposita | |
| MOL001670 | 1. methoxy-3-methyl-9, 2. 10-anthraquinone | 37.83 | 0.21 | Hedyotis diffusa | |
| MOL005435 | 24-Methylcholest-5-enyl-3belta-O-glucopyrAnoside_qt | 37.58 | 0.72 | Dioscorea opposita | |
| MOL005438 | Campesterol | 37.58 | 0.71 | Dioscorea opposita | |
| MOL006774 | Stigmast-7-enol | 37.42 | 0.75 | Prunella vulgaris | |
| MOL011287 | Lucidone A | 37.22 | 0.64 | Ganoderma lucidum | |
| MOL000296 | Hederagenin | 36.91 | 0.75 | Astragalus membranaceus | |
| MOL000358 | Beta-sitosterol | 36.91 | 0.75 | Hedyotis diffusa | |
| MOL000379 | 9,10-dimethoxypterocarpan-3-  O-β-D-glucoside | 36.74 | 0.92 | Astragalus membranaceus | |
| MOL005458 | Dioscoreside C_qt | 36.38 | 0.87 | Dioscorea opposita | |
| MOL000033 | (3S,8S,9S,10R,13R,14S,17R)-10,13-dimethyl-17-[(2R,5S)-5propan-2-yloctan-2-yl]-2,3,4,7,8,9,11,12,14,15,16,17-dodecahydro-1H-cyclopenta[a]phenanthren-3-ol | 36.23 | 0.78 | Astragalus membranaceus | |
| MOL000006 | Luteolin | 36.16 | 0.25 | Ligustrum lucidum | |
| MOL011256 | Ganolucidic acid E | 32.85 | 0.82 | Ganoderma lucidum | |
| MOL011129 | methyl (4R)-4-[(5R,10S,13R,14R,17R)-4,4,10,13,14-pentamethyl-3,7,11,15-tetraoxo-2,5,6,12,16,17-hexahydro-1H-cyclopenta[a]phenanthren-  17-yl]pentanoate | 32.67 | 0.81 | Ganoderma lucidum | |
| MOL000387 | Bifendate | 31.1 | 0.67 | Astragalus membranaceus | |
| MOL001559 | Piperlonguminine | 30.71 | 0.18 | Dioscorea opposita | |
| MOL011270 | (4R)-4-[(5R,7S,10S,13R,14R,17R)-7-hydroxy-3,11,15-triketo-4,4,10,13,14-pentamethyl-1,2,5,6,7,12,16,17-octahydrocyclopenta[a]phenanthren-17-yl]valeric acid | 30.34 | 0.79 | Ganoderma lucidum | |
| MOL011309 | methyl (4R)-4-[(5R,7S,10S,13R,14R,15S,17R)-7,15-dihydroxy-4,4,10,13,14-Pentamethyl-3,11-dioxo-2,5,6,7,12,15,16,17-octahydro-1H-cycloPenta[a]phenanthren-17-yl]pentanoate | 30.19 | 0.81 | Ganoderma lucidum | |
